# Multilocus phylogeny, morphology and taxonomy of *Microdochium (Microdochiaceae)*: insights into evolutionary divergence times and historical biogeography

**DOI:** 10.3897/imafungus.17.191909

**Published:** 2026-06-12

**Authors:** Qiyun Liu, Yuxin Shang, Jinyang Yuan, Zhaoxue Zhang, Congcong Ai, Jiwen Xia, Xiuguo Zhang, Zhuang Li

**Affiliations:** 1 Shandong Provincial Key Laboratory for Biology of Vegetable Diseases and Insect Pests, College of Plant Protection, Shandong Agricultural University, Taian, 271018, China College of Agriculture and Forestry, Linyi University Linyi China; 2 College of Life Sciences, Shandong Normal University, Jinan 250358, China College of Life Sciences, Shandong Normal University Jinan China; 3 College of Agriculture and Forestry, Linyi University, Linyi, Shandong, 276000, China College of Plant Protection, Shandong Agricultural University Taian China

**Keywords:** Ancestral state reconstruction, *
Microdochium
*, molecular clock, morphology, multilocus phylogeny, taxa

## Abstract

*Microdochiaceae* includes many saprobic, endophytic, or pathogenic species, commonly found on plant leaves, seeds, and in soil. Based on samples from four provinces (Fujian, Guangdong, Guangxi, and Hainan) in China, we explored the species diversity, phylogeny, divergence times, and ancestral distribution-determining traits of *Microdochium*. Morphological and multi-gene (ITS, LSU, *rpb2*, and *tub2*) phylogenetic analyses revealed eight new species, three new host records, one new geographical record, and one new combination. The divergence of *Microdochium* emerged with a mean crown age of 49.5 Mya [95% HPD of 38.8–61.2 Mya], which occurred in the Paleogene period. Reconstructing ancestral state in phylogenies (RASP) using the Bayesian Binary Markov chain Monte Carlo (BBM) method to reconstruct the historical biogeography for the genus *Microdochium* indicated that the genus most likely originated in Asia. This study not only advances our understanding of species diversity within *Microdochium*, but also lays the groundwork for *Microdochium* evolutionary research.

## Introduction

*Microdochium*, the type genus of the family *Microdochiaceae*, belongs to the order *Xylariales*, the class *Sordariomycetes* (Hernández-Restrepo et al. 2016; Absalan et al. 2025; Shang et al. 2025). *Microdochium* species are important plant pathogens, particularly on grasses and cereals. In cold to temperate regions, *Microdochium nivale* and *M. majus* cause ‘Microdochium patch’, an economically important disease of wheat and barley (Von Arx 1987; Glynn et al. 2005; Jewell and Hsiang 2013). *Microdochium* species, also been identified as endophytes and saprobes, are widely distributed in plant tissues (including roots, leaves, and seeds), as well as in diverse environmental settings (air, water, soil, and organic litter) (Mandyam et al. 2010; Shen et al. 2014; Zhang et al. 2017; Das et al. 2020). Zhang et al. (2023a) collected *M. phyllosaprophyticum* as a saprobe from saprophytic leaves. *Microdochium hongkuii* was isolated as an endophyte from *Hyparrhenia newtonii* by Yan and Zhang (2024). In recent years, many taxonomists have increased the known diversity of *Microdochium* (Zhang et al. 2024; Shang et al. 2025). Currently, 80 records are listed for this genus in the Index Fungorum (http://www.indexfungorum.org/, 13 May 2026).

*Microdochium* was established with the type species *M. phragmitis*, found on *Phragmites australis* by Sydow (1924) in Germany. The genus is characterized by its globose, erumpent stromata, verticillate conidiophores, holoblastic, discrete, small papillate conoid conidiogenous cells and conidia that are dry or in slimy mass, unicellular or multiseptate, hyaline, smooth, lunate, falcate, fusiform, filiform, obovoid or subpyriform, straight or curved, with rounded apex and flattened base (Sydow 1924; Hernández-Restrepo et al. 2016). *Monographella* and *Microdochium* were both described in the 1924 volume of Annales Mycologici (Petrak 1924; Sydow 1924). *Monographella* was considered the sexual morph of *Microdochium* (Zhang et al. 2015; Hernández-Restrepo et al. 2016; Zhang et al. 2017). *Microdochium* has more species, is more commonly encountered, and its name is more frequently used in the literature; consequently, it was protected under the implementation of the ‘one fungus one name’ principle (Hawksworth et al. 2011; Hernández-Restrepo et al. 2016). Previous studies placed *Idriella* within *Helotiales* (Kimbrough and Atkinson 1972) and associated *Microdochium* with *Amphisphaeriaceae* (Parkinson et al. 1981; Samuels and Hallett 1983; Von Arx 1984; Jaklitsch and Voglmayr 2011). Based on phylogenetic analyses, Hernández-Restrepo et al. (2016) proposed *Microdochiaceae* to accommodate *Microdochium*, *Idriella*, and *Selenodriella*. Hernández-Restrepo et al. (2022) isolated four strains (three as *Gyrothrix verticiclada*, one as *Gyrothrix hughesii*) that clustered in a clade distant from *Gyrothrix* but close to *Selenodriella* in *Microdochiaceae*, leading them to resurrect the genus *Peglionia* (Goidanich 1934). Crous et al. (2023) described *Xenoidriella* as a genus distinct from *Neoidriella desertorum* and *Guayaquilia*, based on differences in conidial septation and chlamydospore production. Zhang et al. (2024) established a new genus, *Macroidriella*, characterized by lunate and curved conidia, which differs from *Microdochium* that produces elliptical conidia. However, the conidial morphology of *Microdochium* is known to include lunate and curved forms, as well as other shapes such as fusiform, falcate, and filiform (Sydow 1924; Hernández-Restrepo et al. 2016). Consequently, the lunate and curved conidia alone do not justify the establishment of *Macroidriella* as a distinct genus, raising questions about its taxonomic validity. Currently, *Microdochiaceae* comprises 6 genera, including *Peglionia* Goid., *Idriella* P.E. Nelson & S. Wilh., *Macroidriella* Z.X. Zhang, J.W. Xia & X.G. Zhang, *Microdochium* Syd., *Selenodriella* R.F. Castañeda & W.B. Kendr, *Xenoidriella* Crous (Hyde et al. 2024).

The molecular clock methodology, using fossil records or geological events as calibration points within a phylogenetic tree framework, is used to estimate divergence times among different taxonomic groups (Avise and Johns 1999; Lockwood et al. 2013; Zhao et al. 2016, 2017; Zhang et al. 2023b; Mu et al. 2024; Zhang et al. 2025). Fungal taxonomic ranks should align with molecular divergence times and the geological ages they represent (Hennig 1966; Zhao et al. 2016; Wang et al. 2025). Based on such analyses, current models indicate that the crown ages of *Amphisphaeriales* and *Xylariales* are estimated at ∼141 Mya (95% HPD = 225.09–88.61 Mya) and ∼153 Mya (95% HPD = 236.75–99.03 Mya), respectively (Samarakoon et al. 2016; Hyde et al. 2020; Chen et al. 2023).

In this study, the morphology, phylogeny, divergence times, and historical biogeography of *Microdochium* are investigated. In addition, eight new species (*M. bambusicola* sp. nov., *M. guangdongense* sp. nov., *M. ledongense* sp. nov., *M. microstegii* sp. nov., *M. nigrum* sp. nov., *M. setariae* sp. nov., *M. viridis* sp. nov., and *M. vulgaris* sp. nov.), three new host records (*M. guizhouensis*, *M. miscanthi*, and *M. sinense*), one new geographical record (*M. nannuoshanense*), and one new combination (*M. danzhouense***nom. nov**.) are described and illustrated. Based on host association, geographic distribution, morphology, phylogenetic placement, and divergence times, we synonymize *Macroidriella* with *Microdochium*. These findings significantly contribute to understanding the species diversity within the genus *Microdochium* and provide critical insights into the evolutionary processes in this fungal group.

## Materials and methods

### Isolation and morphological studies

From 2023 to 2025, healthy and diseased leaves of specific host plants were collected in Fujian, Guangdong, Guangxi, and Hainan Provinces. After collection, the samples were labeled chronologically with collection time, location, and host plant species, then photographed and documented. Using the tissue isolation technique, we obtained pure cultures. For each sample, 25 mm^2^ leaf lesion fragments were excised from the margins of the tissues. The surface sterilization process consisted of the following sequential steps: immersing in 75% ethanol for 1 min, rinsing with sterile distilled water for 30 s, treating with 5% sodium hypochlorite for 30 s, and a final three rinses with sterile distilled water. The sterilized fragments were blotted dry on sterile filter paper and transferred to PDA plates for incubation at 25 °C in the dark for 3 days. Hyphal tips were picked from the periphery of the colonies on PDA and inoculated onto new PDA plates for morphological examination. The colony morphology, pigmentation, and growth rates of the isolates were recorded. The surfaces of the PDA plates (above and reverse) were imaged after one week using a Canon Powershot G7X digital camera (Canon, Tokyo, Japan). Micromorphological features were observed using an Olympus SZ61 stereomicroscope and an Olympus BX53 microscope equipped with differential interference contrast (DIC). Both microscopes were fitted with BioHD-A20c color digital cameras for imaging the fungal structures. Microstructures were measured randomly using Digimizer software v5.6.0 (https://www.digimizer.com), and their av. was calculated. All fungal strains were preserved in 10% sterilized glycerol at 4 °C for future studies. Voucher specimens are preserved in the Herbarium of the Department of Plant Pathology, Shandong Agricultural University, Tai’an, China (**HSAUP**). Corresponding ex-type living cultures were preserved in two culture collections: the Shandong Agricultural University Culture Collection (**SAUCC**) and the China General Microbiological Culture Collection Center (**CGMCC**). The taxonomic data have been deposited in the Fungal Names repository (https://nmdc.cn/fungalnames/). The genus abbreviations used in this study are as follows: *I.* = *Idriella*, *M.*= *Microdochium*, *P.* = *Peglionia*, *S.* = *Selenodriella*.

### DNA extraction and sequencing

Total genomic DNA was extracted from fresh fungal mycelia grown on potato dextrose agar (PDA) for 7 days using cetyltrimethylammonium bromide (CTAB) or a commercial kit (DC112-C7, VAMNE Magnetic Bacteria/Fungi DNA Extraction Kit (Prepackaged), Vazyme Biotech Co., Ltd). For multi-locus phylogenetic analysis, the following four genetic regions were amplified and sequenced: the internal transcribed spacer (ITS) region, the large subunit (LSU) of the rRNA gene, the RNA polymerase second largest subunit (*rpb2*) gene, and the partial beta-tubulin (*tub2*) gene. The primer information and corresponding references are provided in Table 1. PCR was performed in a 20 μL reaction mixture containing 10 μL of 2 × Hieff Canace® Plus PCR Master Mix (Yeasen Biotechnology, Cat. No. 10154ES03), 0.5 μL each of forward and reverse primers (10 μM; TsingKe, Qingdao, China), 1 μL of template genomic DNA, and nuclease-free water to the final volume. Amplified products were electrophoresed on a 1% agarose gel (Cat. Nos. RM02852, ABclonal Biotechnology Co., Ltd., Wuhan, China) stained with GelStain (GS101; TransGen Biotech, China) and visualized under UV light. Target bands were excised and purified using a gel extraction kit (DM1200, Beijing Solarbio Science & Technology Co., Ltd.). Purified PCR products were sequenced by Youkang Company Limited (Zhejiang, China). Sequence data were analyzed using MEGA v.7.0 (Kumar et al. 2016). All newly generated sequences were deposited in GenBank (https://www.ncbi.nlm.nih.gov/) (Table 2).

**Table 1.** Gene loci and corresponding PCR primers and programs used in this study.

Locus	PCR primers	Sequence (5’ – 3’)	PCR cycles	References
ITS	ITS5	GGA AGT AAA AGT CGT AAC AAG G	(94 °C: 30 s, 55 °C: 30 s, 72 °C: 45 s) × 29 cycles	(White et al. 1990)
	ITS4	TCC TCC GCT TAT TGA TAT GC		
LSU	LR0R	GTA CCC GCT GAA CTT AAG C	(94 °C: 30 s, 48 °C: 50 s, 72 °C: 1 min 30 s) × 35 cycles	(Vilgalys and Hester 1990)
	LR5	TCC TGA GGG AAA CTT CG		
*rpb2*	RPB2-5F2	GGG GWG AYC AGA AGA AGG C	(94 °C: 45 s, 60 °C: 45 s, 72 °C: 2 min) × 5 cycles, (94 °C: 45 s, 54 °C: 45 s, 72 °C: 2 min) × 30 cycles	(Liu et al. 1999; Sung et al. 2007)
	RPB2-7CR	CCC ATR GCT TGY TTR CCC AT		
*tub2*	Btub526-F	CGA GCG YAT GAG YGT YTA CTT	(95 °C: 30 s, 56 °C: 30 s, 72 °C: 45 s) × 35 cycles	(Jewell and Hsiang 2013)
	Btub1332-R	TCA TGT TCT TGG GGT CGA A		

**Table 2.** GenBank accession numbers of the taxa used in phylogenetic reconstruction.

Species	Strains	ITS	LSU	*rpb2*	*tub2*	References
*Idriella chlamydospora*	CGMCC 3.20778*	OL897016	OL897058	NA	ON569069	(Zhang et al. 2023c)
*I. chlamydospora*	GZUIFR 21.922	OL897017	OL897059	NA	ON569070	(Zhang et al. 2023c)
*I. lunata*	CBS 204.56*	KP859044	KP858981	NA	NA	(Hernández-Restrepo et al. 2016)
*I. lunata*	CBS 177.57	KP859043	KP858980	NA	NA	(Hernández-Restrepo et al. 2016)
*I. multiformispora*	CGMCC 3.20779*	OL897018	OL897060	ON568988	ON569071	(Zhang et al. 2023c)
*I. multiformispora*	GZUIFR 21.924	OL897019	OL897061	ON568989	ON569072	(Zhang et al. 2023c)
*I. multiformispora*	GZUIFR 21.925	OL897020	OL897062	ON568990	ON569073	(Zhang et al. 2023c)
*Microdochium albescens*	CBS 243.83	KP858994	KP858930	KP859103	KP859057	(Hernández-Restrepo et al. 2016)
*Microdochium albescens*	CBS 291.79	KP858996	KP858932	KP859105	KP859059	(Hernández-Restrepo et al. 2016)
*M. australe*	SAUCC 6322-5-1*	PP695312	PP702043	PP716780	PP716787	(Zhang et al. 2024)
*M. australe*	SAUCC 6151-1	PP695313	PP702044	PP716779	PP716788	(Zhang et al. 2024)
*M. australiana*	SAUCC 6340-2-6 = CGMCC 3.28622*	PQ807110	PV609100	PV975978	PV686755	(Shang et al. 2025)
*M. australiana*	SAUCC 8723-2	PQ807111	PV609101	PV975979	PV686756	(Shang et al. 2025)
*M. baishamenense*	SAUCC 8129-1 = CGMCC 3.28625*	PQ807114	PV609104	PV975982	PV686759	(Shang et al. 2025)
*M. baishamenense*	SAUCC 7263-1	PQ807115	PV609105	PV975983	PV686760	(Shang et al. 2025)
***M. bambusicola***	SAUCC 7623-2 = CGMCC 3.29432*	**PX569617**	**PX578006**	**PX705309**	**PX705279**	**This study**
***M. bambusicola***	**SAUCC 7623-2A**	**PX569618**	**PX578007**	**PX705308**	**PX705278**	**This study**
*M. bambusae*	SAUCC 1862-1*	OR702567	OR702576	OR715785	PP445175	(Zhang et al. 2023a)
*M. bambusae*	SAUCC 1866-1	OR702568	OR702577	OR715786	PP445176	(Zhang et al. 2023a)
*M. bambusarum*	SAUCC 7611-3 = CGMCC 3.28624*	PQ807112	PV609102	PV975980	PV686757	(Shang et al. 2025)
*M. bambusarum*	SAUCC 6699-4	PQ807113	PV609103	PV975981	PV686758	(Shang et al. 2025)
*M. bambusina*	SAUCC 7531-3 = CGMCC 3.28623*	PQ807108	PV609098	PV975976	PV686753	(Shang et al. 2025)
*M. bambusina*	SAUCC 7638-2	PQ807109	PV609099	PV975977	PV686754	(Shang et al. 2025)
*M. bolleyi*	CBS 540.92	KP859010	KP858946	KP859119	KP859073	(Hernández-Restrepo et al. 2016)
*M. buffelskloofinum*	SA1SD *	PP791437.1	PP791465.1	PP780615.1	PP780639.1	(Crous et al. 2024)
*M. buffelskloofinum*	CPC 47528	PP791438.1	PP791466.1	PP780616.1	PP780640.1	(Crous et al. 2024)
*M. chrysanthemoides*	CGMCC 3.17929*	KU746690	KU746736	KY883244	NA	(Zhang et al. 2017)
*M. chrysanthemoides*	CGMCC 3.17930	KU746689	KU746735	KY883245	NA	(Zhang et al. 2017)
*M. chrysopogonis*	GDMCC 3.683*	MT988022	MT988024	MW002444	MW002441	(Lu et al. 2023)
*M. chrysopogonis*	LNU-196	MT988020	MT988023	MW002445	MW002442	(Lu et al. 2023)
*M. chuxiongense*	YFCC 8794*	OK586161	OK586160	OK584019	OK556901	(Tang et al. 2022)
*M. citrinidiscum*	CBS 109067*	KP859003	KP858939	KP859112	KP859066	(Hernández-Restrepo et al. 2016)
*M. colombiense*	CBS 624.94*	KP858999	KP858935	KP859108	KP859062	(Hernández-Restrepo et al. 2016)
***M. danzhouense***	SAUCC 6792-1*	**PP716851**	**PP716512**	**PP729053**	**PP729058**	**This study**
***M. danzhouense***	**SAUCC 6792-2**	**PP716852**	**PP716513**	**PP729054**	**PP729059**	**This study**
***M. danzhouense***	**SAUCC 6792-5**	**PP716853**	**PP716514**	**PP729055**	**PP729060**	**This study**
***M. danzhouense***	**SAUCC 6113-1**	**PP716854**	**PP716515**	**PP729056**	**PP729061**	**This study**
***M. danzhouense***	**SAUCC 6113-3**	**PP716855**	**PP716516**	**PP729057**	**PP729062**	**This study**
*M. dawsoniorum*	BRIP 65649*	MK966337	NA	NA	NA	(Crous et al. 2020)
*M. fisheri*	CBS 242.90*	KP859015	KP858951	KP859124	KP859079	(Hernández-Restrepo et al. 2016)
*M. gongcheniae*	YNE01155	PP111925	PP111932	NA	PP112585	(Yan and Zhang 2024)
*M. gongcheniae*	GDMCC3.1048 = YNE01164*	PP111926	PP111933	NA	PP112586	(Yan and Zhang 2024)
*M. graminearum*	CGMCC 3.23525*	OP103966	OP104016	OP236027	NA	(Gao et al. 2022)
*M. graminearum*	CGMCC 3.23524	OP103965	OP104015	OP236026	NA	(Gao et al. 2022)
*M. graminis*	GDMCC3.1049 *	PP111928	PP111935	PP112593	PP112588	(Yan and Zhang 2024)
*M. graminis*	B13	HQ696038	NA	NA	NA	(Shen et al. 2014)
*M. graminis*	PE110	JX875927	NA	NA	NA	(Shen et al. 2012)
***M. guangdongense***	SAUCC 17239-1 = CGMCC 3.29429*	**PX569636**	**PX578025**	**PX705328**	**PX705298**	**This study**
***M. guangdongense***	**SAUCC 17239-1A**	**PX569637**	**PX578026**	**PX705327**	**PX705297**	**This study**
*M. guizhouensis*	GUCC 25–0012*	NA	NA	PV505431	PV505433	(Hongsanan et al. 2025)
*M. guizhouensis*	GUCC 25–0013	NA	NA	PV505432	PV505434	(Hongsanan et al. 2025)
***M. guizhouensis***	**SAUCC 11159-1**	**PX569620**	**PX578009**	**PX705311**	**PX705281**	**This study**
*M. hainanense*	SAUCC 210782	OM956296	OM959324	OM981154	OM981147	(Liu et al. 2022)
*M. hainanense*	SAUCC 210781*	OM956295	OM959323	OM981153	OM981146	(Liu et al. 2022)
*M. hongkuii*	YNE00384	PP111922	PP111929	PP112589	PP112582	(Yan and Zhang 2024)
*M. hongkuii*	GDMCC3.1079 = YNE00483*	PP111923	PP111930	PP112590	PP112583	(Yan and Zhang 2024)
*M. hongkuii*	N115	MK304137	NA	NA	NA	From NCBI
*M. indocalami*	SAUCC 1016*	MT199884	MT199878	MT510550	MT435653	(Huang et al. 2020)
*M. insulare*	BRIP 75114a	OQ917075	OQ892168	OQ889560	NA	(Tan and Shivas 2023a)
*M. jianfenglingense*	SAUCC 1862-2*	PP702394	PP711783	PP716793	PP716799	(Li et al. 2024)
*M. jianfenglingense*	SAUCC 1862-5	PP702395	PP711784	PP716794	PP716800	(Li et al. 2024)
***M. ledongense***	SAUCC 14499-3 = CGMCC 3.29650*	**PX569628**	**PX578017**	**PX705319**	**PX705289**	**This study**
***M. ledongense***	**SAUCC 16723-1**	**PX569629**	**PX578018**	**PX705320**	**PX705290**	**This study**
***M. ledongense***	**SAUCC 16889-1**	**PX569630**	**PX578019**	**PX705321**	**PX705291**	**This study**
***M. ledongense***	**SAUCC 16889-3**	**PX569631**	**PX578020**	**PX705322**	**PX705292**	**This study**
***M. ledongense***	**SAUCC 16957-1**	**PX569632**	**PX578021**	**PX705323**	**PX705293**	**This study**
***M. ledongense***	**SAUCC 17376-5**	**PX569640**	**PX578029**	**PX705331**	**PX705301**	**This study**
*M. lycopodinum*	CBS 146.68	KP858993	KP858929	KP859102	KP859056	(Hernández-Restrepo et al. 2016)
*M. lycopodinum*	CBS 122885*	KP859016	KP858952	KP859125	KP859080	(Hernández-Restrepo et al. 2016)
*M. maculosum*	COAD 3358*	OK966954	Ok966953	NA	NA	(Crous et al. 2021b)
*M. majus*	CBS 741.79	KP859001	KP858937	KP859110	KP859064	(Hernández-Restrepo et al. 2016)
***M. microstegii***	**SAUCC 14103-3**	**PX569625**	**PX578014**	**PX705316**	**PX705286**	**This study**
***M. microstegii***	SAUCC 14288-1 = CGMCC 3.29421*	**PX569626**	**PX578015**	**PX705317**	**PX705287**	**This study**
***M. microstegii***	**SAUCC 18044-6**	**PX569643**	**PX578032**	**PX705334**	**PX705304**	**This study**
*M. miscanthi*	SAUCC 211092*	OM956214	OM957532	OM981148	OM981141	(Liu et al. 2022)
*M. miscanthi*	SAUCC 211093	OM956215	OM957533	OM981149	OM981142	(Liu et al. 2022)
***M. miscanthi***	**SAUCC 7610-2**	**PX569616**	**PX578005**	**PX705307**	**PX705277**	**This study**
***M. miscanthi***	**SAUCC 7638-2A**	**PX569619**	**PX578008**	**PX705310**	**PX705280**	**This study**
***M. miscanthi***	**SAUCC 13968-2**	**PX569621**	**PX578010**	**PX705312**	**PX705282**	**This study**
***M. miscanthi***	**SAUCC 14090-2**	**PX569624**	**PX578013**	**PX705315**	**PX705285**	**This study**
*M. musae*	CBS 143500*	MH107895	MH107942	MH108003	NA	(Crous et al. 2018)
*M. musae*	CBS 143499	MH107894	MH107941	NA	NA	(Crous et al. 2018)
*M. nannuoshanense*	SAUCC 2450-1*	OR702569	OR702578	OR715787	PP445177	(Zhang et al. 2023a)
*M. nannuoshanense*	SAUCC 2450-3	OR702570	OR702579	OR715788	PP445178	(Zhang et al. 2023a)
***M. nannuoshanense***	**SAUCC 13988-3**	**PX569622**	**PX578011**	**PX705313**	**PX705283**	**This study**
*M. neoqueenslandicum*	CBS 445.95	KP858997	KP858933	KP859106	KP859060	(Hernández-Restrepo et al. 2016)
*M. neoqueenslandicum*	CBS 108926*	KP859002	KP858938	KP859111	KP859065	(Hernández-Restrepo et al. 2016)
***M. nigrum***	SAUCC 17349-2 = CGMCC 3.29428*	**PX569638**	**PX578027**	**PX705330**	**PX705300**	**This study**
***M. nigrum***	**SAUCC 17349-2A**	**PX569639**	**PX578028**	**PX705329**	**PX705299**	**This study**
*M. nivale*	CBS 116205*	KP859008	KP858944	KP859117	KP859071	(Hernández-Restrepo et al. 2016)
*M. nivale*	CBS 288.50	MH856626	MH868135	NA	NA	(Vu et al. 2019)
*M. nivale* var majus	CBS 177.29	MH855031	MH866500	NA	NA	(Vu et al. 2019)
*M. novae-zelandiae*	CPC 29376*	LT990655	NA	LT990641	LT990608	(Marin-Felix et al. 2019)
*M. novae-zelandiae*	CPC 29693	LT990656	NA	LT990642	LT990609	(Marin-Felix et al. 2019)
*M. oryzicola*	MFLUCC 24-0509*	PV241406	PV241407	PV275683	NA	(Absalan et al. 2025)
*M. paspali*	HK-ML-1371	KJ569509	NA	NA	KJ569514	(Zhang et al. 2015)
*M. paspali*	CBS 138620*	KJ569513	NA	NA	KJ569518	(Zhang et al. 2015)
*M. phragmitis*	CBS 285.71*	KP859013	KP858949	KP859122	KP859077	(Hernández-Restrepo et al. 2016)
*M. phragmitis*	CBS 423.78	KP859012	KP858948	KP859121	KP859076	(Hernández-Restrepo et al. 2016)
*M. phyllosaprophyticum*	SAUCC 3583-1*	OR702571	OR702580	OR715789	PP445179	(Zhang et al. 2023a)
*M. phyllosaprophyticum*	SAUCC 3583-6	OR702572	OR702581	OR715790	PP445180	(Zhang et al. 2023a)
*M. poae*	CGMCC 3.19170*	MH740898	NA	MH740906	MH740914	(Liang et al. 2019)
*M. poae*	LC 12115	MH740901	NA	MH740909	MH740917	(Liang et al. 2019)
*M. poae*	LC 12116	MH740902	NA	MH740910	MH740918	(Liang et al. 2019)
*M. ratticaudae*	BRIP 68298*	MW481661	MW481666	MW626890	NA	(Crous et al. 2021a)
*M. rhopalostylidis*	CBS 145125*	MK442592	MK442532	MK442667	NA	(Crous et al. 2019)
*M. salmonicolor*	KCTC 56427	MK836110	MK836108	NA	NA	(Das et al. 2020)
*M. seminicola*	CBS 139951*	KP859038	KP858974	KP859147	KP859101	(Hernández-Restrepo et al. 2016)
*M. seminicola*	CPC 26001	KP859025	KP858961	KP859134	KP859088	(Hernández-Restrepo et al. 2016)
*M. seminicola*	DAOM 250161	KP859034	KP858970	KP859143	KP859097	(Hernández-Restrepo et al. 2016)
***M. setariae***	**SAUCC 17215-3**	**PX569633**	**PX578022**	**PX705324**	**PX705294**	**This study**
***M. setariae***	SAUCC 18044-7 = CGMCC 3.29420*	**PX569644**	**PX578033**	**PX705335**	**PX705305**	**This study**
***M. setariae***	**SAUCC 18057-1**	**PX569645**	**PX578034**	**PX705336**	**PX705306**	**This study**
*M. shilinense*	CGMCC 3.23531*	OP103972	OP104022	NA	OP242834	(Gao et al. 2022)
*M. sichuanense*	KUNCC23-13008*	OQ616510	OQ616434	OQ623473	NA	(Dissanayake et al. 2023)
*M. sinense*	SAUCC 211097*	OM956289	OM959225	OM981151	OM981144	(Liu et al. 2022)
*M. sinense*	SAUCC 211098	OM956290	OM959226	OM981152	OM981145	(Liu et al. 2022)
***M. sinense***	**SAUCC 14457-1**	**PX569627**	**PX578016**	**PX705318**	**PX705288**	**This study**
*M. sorghi*	CBS 691.96	KP859000	KP858936	KP859109	KP859063	(Hernández-Restrepo et al. 2016)
*M.* sp.	YNE01043	PP111924	PP111931	PP112591	PP112584	(Yan and Zhang 2024)
*M.* sp.	YNE01771	PP111927	PP111934	PP112592	PP112587	(Yan and Zhang 2024)
*M.* sp.	ZJ40	KJ572190	NA	NA	NA	From NCBI
*M. streetiae*	BRIP 74742a*	OR947072	OR947079	NA	NA	(Tan and Shivas 2023b)
*M. streetiae*	BRIP 74752a	OR947071	OR947078	NA	NA	(Tan and Shivas 2023b)
*M. tainanense*	CBS 269.76*	KP859009	KP858945	KP859118	KP859072	(Hernández-Restrepo et al. 2016)
*M. tainanense*	CBS 270.76	KP858995	KP858931	KP859104	KP859058	(Hernández-Restrepo et al. 2016)
*M. trichocladiopsis*	CBS 623.77 = CBS H-22137*	KP858998	KP858934	KP859107	KP859061	(Hernández-Restrepo et al. 2016)
*M. triticicola*	RR 241*	AJ748691	NA	NA	NA	(Kwasna and Bateman 2007)
***M. viridis***	**SAUCC 18044-2A**	**PX569641**	**PX578031**	**PX705332**	**PX705302**	**This study**
***M. viridis***	SAUCC 18044-2 = CGMCC 3.29434*	**PX569642**	**PX578030**	**PX705333**	**PX705303**	**This study**
***M. vulgaris***	SAUCC 17227-1 = CGMCC 3.29422*	**PX569634**	**PX578023**	**PX705326**	**PX705296**	**This study**
***M. vulgaris***	**SAUCC 17227-1A**	**PX569635**	**PX578024**	**PX705325**	**PX705295**	**This study**
*M. yunnanense*	SAUCC 1011*	MT199881	MT199875	MT510547	MT435650	(Huang et al. 2020)
*M. yunnanense*	SAUCC 1012	MT199882	MT199876	MT510548	MT435651	(Huang et al. 2020)
*M. yunnanense*	SAUCC1015	MT199883	MT199877	MT510549	MT435652	(Huang et al. 2020)
*M. yunnanense*	SAUCC1018	MT199886	MT199880	NA	MT435655	(Huang et al. 2020)
*Peglionia falcata*	GUCC 23–0042*	PP295269	PP314032	PP396044	NA	(Fu et al. 2024)
*P. falcata*	GUCC 23–0043	PP295270	PP314033	PP396045	NA	(Fu et al. 2024)
*P. falcata*	GUCC 23–0044	PP295271	PP349828	PP396046	NA	(Fu et al. 2024)
*Selenodriella cubensis*	CBS 683.96	KP859053	KP858990	NA	NA	(Hernández-Restrepo et al. 2016)
*S. fertilis*	CBS 772.83	KP859055	KP858992	NA	NA	(Hernández-Restrepo et al. 2016)
*Xenoidriella cinnamomic*	HPC 3839 = CPC 43130 = CBS 149458	OQ628471	OQ629053	OQ627937	NA	(Crous et al. 2023)
*Muscodor fengyangensis*	CGMCC 2862*	HM034856	HM034859	HM034849	HM034843	(Zhang et al. 2010)
*Muscodor thailandicus*	MFLUCC 17–2669*	MK762707	MK762714	MK791283	MK776960	(Samarakoon et al. 2020; Cedeno-Sanchez et al. 2023)

Notes: Species established in this study are shown in bold. Those marked “*” in the table are represented as ex-type or ex-epitype strains. NA: not available.

### Phylogenetic analyses

**Figure 1.**
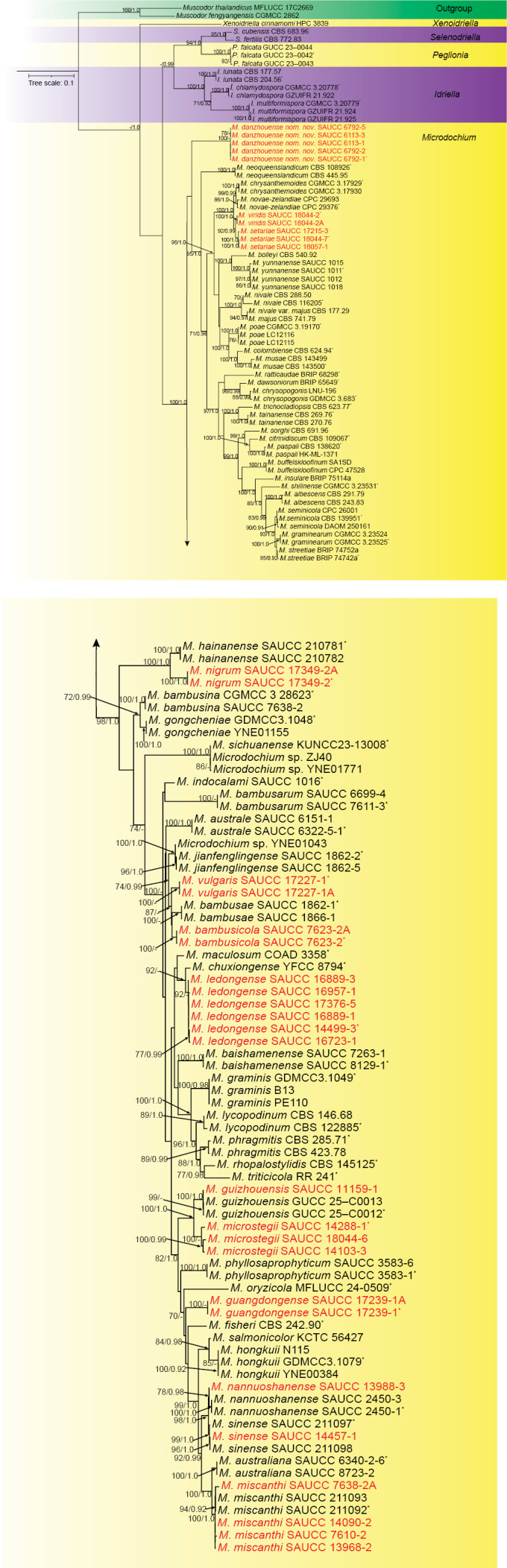
Phylogram of the family *Microdochiaceae* based on a concatenated ITS, LSU, *rpb2*, and *tub2* sequence alignment, with *Muscodor fengyangensis* (CGMCC 2862) and *Muscodor thailandicus* (MFLUCC 17–2669) serving as outgroups. The Bayesian inference posterior probability (left, BIPP ≥ 0.90) and the maximum likelihood bootstrap value (right, MLBV ≥ 70%) are shown as BIPP/MLBV above the nodes. Those marked “*” in the tree are represented as ex-type or ex-epitype strains. Strains isolated in this study are indicated in red. Furthermore, four single gene trees were individually evaluated for *Microdochiaceae* (Supplementary Information 2).

All sequences were aligned using the MAFFT v.7 online service and subsequently manually corrected in MEGA v.7.0 (Katoh et al. 2019). The concatenated ITS, LSU, *rpb2*, and *tub2* sequences were used for phylogenetic analyses. Maximum likelihood (ML) analysis was conducted in RAxML-HPC2 on XSEDE v.8.2.12 with 1000 rapid bootstrap replicates model using default parameters. Bayesian inference (BI) analysis was performed in MrBayes v.3.2.7a on Linux, employing Markov chain Monte Carlo (MCMC) algorithms; the best-fit evolutionary model for each partition was determined using MrModeltest v.2.3 (Nylander 2004). The phylogenetic trees were visualized and optimized using the Interactive Tree of Life (ITOL) platform (https://itol.embl.de/), and the final layout was refined in Adobe Illustrator CS6 (Adobe Systems Inc., USA). The isolates in this study are marked in red in the phylogenetic tree (Fig. 1).

### Pairwise homoplasy index (PHI) test

The pairwise homoplasy index (PHI) test was performed using the SplitsTree App to assess potential recombination events among closely related phylogenetic species (Bruen et al. 2006; Huson and Bryant 2024). The analysis used a concatenated dataset of four loci (ITS, LSU, *rpb2*, and *tub2*), and a Φw-statistic below 0.05 (*p*-value < 0.05) revealed significant evidence of recombination. To further elucidate relationships among closely related taxa, split graphs were constructed using the Log-Det transformation and split decomposition methods, providing a clear and intuitive visualization of phylogenetic relationships.

### Divergence time estimation

The ITS + LSU + *rpb2* + *tub2* concatenated sequence dataset with 59 specimens was used to infer the divergence times of species in the genus *Microdochium*, with divergence times estimated using the BEAST v.2.7.7 (Bouckaert et al. 2014), the complete generated nucleotide dataset. The best models of evolution were selected with MrModeltest v.2.3 (Nylander 2004). The XML (Extensible Markup Language) file was generated in BEAUti v.2.7.7. The ITS + LSU + *rpb2* + *tub2* concatenated sequence dataset was set as different partitions, with substitution and clock models unlinked while the trees were linked. Divergence time and corresponding CIs were estimated with a Relaxed Clock Log Normal and the Yule speciation prior (Drummond et al. 2006; Lepage et al. 2007; Gernhard 2008). Three fossil calibration points were used: *Xylaria antiqua* (Poinar Jr 2014) and *Paleopyrenomycites devonicus* (Taylor et al. 1999, 2005), representing the divergence time of *Xylariaceae*, and *Pezizomycotina* were selected for calibration, respectively. Consistent with previous studies (Samarakoon et al. 2016; Hyde et al. 2020; Chen et al. 2023), the crown age of *Xylariales* is estimated at approximately 153 Mya. The offset ages with a gamma-distributed prior (scale = 20 and shape = 1) were set as 25, 153, and 400 Mya for *Xylariaceae*, *Xylariales*, and *Pezizomycotina*, respectively. Four independent Markov chain Monte Carlo (MCMC) chains of 100 million generations were conducted and parameters were sampled every 10,000 generations. The resulting log file was evaluated for convergence and stationarity with Tracer v.1.7.2 (ESS ≥ 200 was considered as convergence) (Rambaut et al. 2018). Afterwards, the first 10% trees representing the starting and unreliable results were discarded as burn-in and a maximum clade tree was created and the 95% highest posterior density (HPD) intervals were calculated by TreeAnnotator v.2.6.7. The posterior probability (PP) ≥ 0.90 was considered as significantly supported.

### Inferring historical biogeography

The Reconstructing Ancestral State in Phylogenies (RASP v.4.3) was used to reconstruct the historical biogeography for the genus *Microdochium* with Bayesian Binary Markov chain Monte Carlo (BBM) method using 10 million generations. Sampling was performed every 1000 generations, with the first 10% of samples discarded as burn-in (Yu et al. 2015, 2020). The geographic distributions for *Microdochium* were identified in five areas: (A) Asia, (B) Europe, (C) Africa, (D) Oceania, and (E) South America.

## Results

### Phylogenetic analysis

Phylogenetic analysis of *Microdochiaceae* was conducted on 144 isolates, for which strains of *Muscodor fengyangensis* (CGMCC 2862) and *Muscodor thailandicus* (MFLUCC 17–2669) served as the outgroups. The ultimate alignment encompassed 3096 concatenated characters, viz. 1–701 (ITS), 702–1544 (LSU), 1545–2403 (*rpb2*), and 2404–3096 (*tub2*). Amongst these, 2,021 characters were constant, 165 were variable and parsimony-uninformative, and 910 were parsimony-informative. The topologies from the ML and BI analyses agreed; therefore, only the ML tree is shown. Based on the phylogenetic analysis of ITS, LSU, *rpb2*, and *tub2* genes, the 142 strains were grouped into 70 species. BI analysis was performed over 3,420,000 generations resulting in 3,421 trees, 3/4 trees are utilized for computing the posterior probability in the majority rule consensus tree (Fig. 1; first value: BIPP ≥ 0.90 displayed).

### PHI analysis

**Figure 2.**
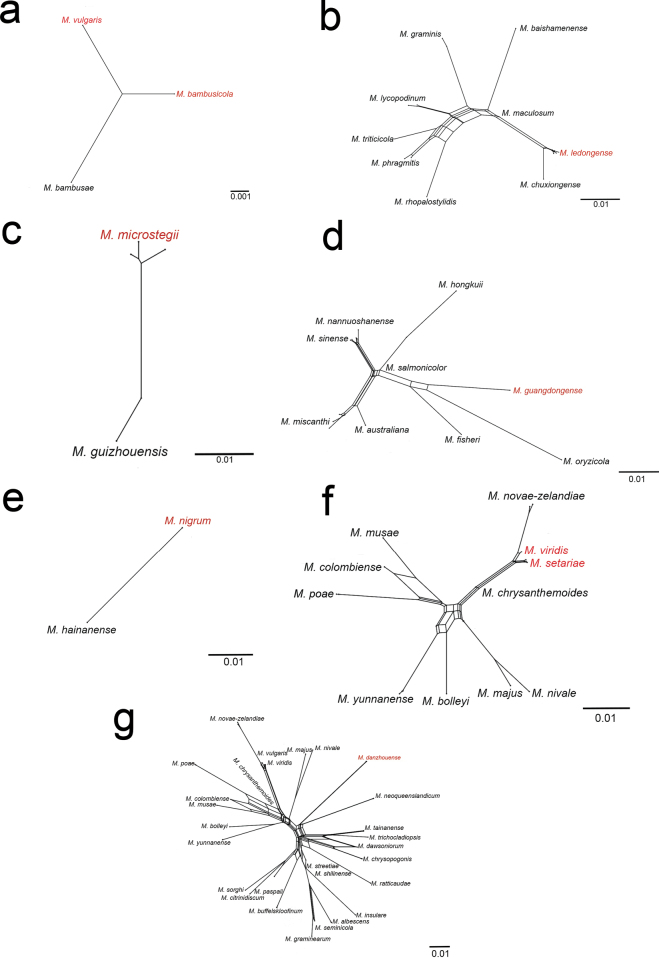
The split graphs of a PHI test result of *Microdochium* species using the LogDet transformation and split decomposition options based on the combined ITS, LSU, *rpb2*, and *tub2* gene loci. a the PHI of *Microdochium vulgaris* sp. nov., *M. bambusicola* sp. nov. with their phylogenetically related isolates or species, *p* = 1.0. b the PHI of *M. ledongense* sp. nov. with their phylogenetically related isolates or species, *p* ≈ 1.0. c the PHI of *M. microstegii* sp. nov. with their phylogenetically related isolates or species, *p* = 1.0. d the PHI of *M. guangdongense* sp. nov. with their phylogenetically related isolates or species, *p* ≈ 1.0. e the PHI of *M. nigrum* sp. nov. with their phylogenetically related isolates or species, *p* = 1.0. f the PHI of *M. viridis* sp. nov., *M. setariae* sp. nov. with their phylogenetically related isolates or species, *p* ≈ 1.0. g the PHI of *M. danzhouense* sp. nov. with their phylogenetically related isolates or species, *p* = 1.0. Strains isolated in this study are indicated in red.

To validate species delineation within *Microdochium*, PHI analysis was conducted. Seven clades, comprising both established and newly proposed species, were selected for testing. The PHI test indicated no significant evidence of genetic recombination within these clades (a, *p* = 1.0; b, *p* ≈ 1.0; c, *p* = 1.0; d, *p* ≈ 1.0; e, *p* = 1.0; f, *p* ≈ 1.0; g, *p* = 1.0 Fig. 2). These results provide robust support for the genetic distinctness of the eight new species proposed in this study, confirming their validity as separate taxonomic entities.

### Divergence time estimation

**Figure 3.**
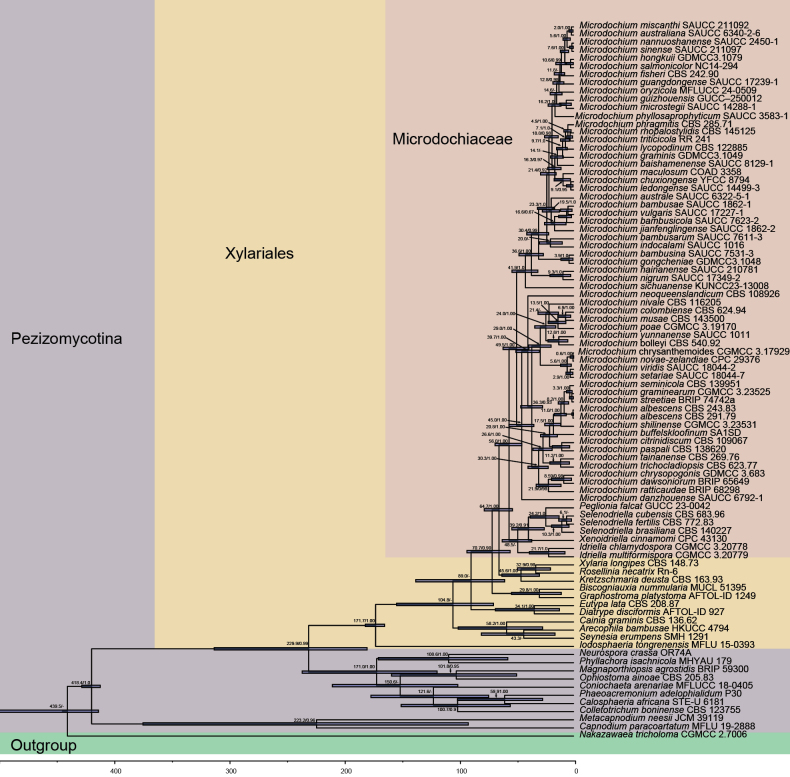
Divergence time estimation of *Microdochium* inferred from molecular clock analyses based on four-locus dataset of ITS, LSU, *rpb2*, and *tub2* regions. The 95% highest posterior density (HPD) of estimated divergence times of divergence time estimates is indicated by horizontal blue bars. The mean divergence times of each node and Bayesian posterior probabilities (BPP) not less than 0.90 are shown at the internodes, respectively. Scale in millions of years (Mya).

Divergence time estimation was performed as detailed in the Methods section, and the divergence time of the main *Microdochium* clade emerged with a mean stem age of 56.0 Mya [95% HPD of 44.9–68.1 Mya] and a mean crown age of 49.5 Mya [95% HPD of 38.8–61.2 Mya], which belongs to the Paleogene period (Fig. 3).

### Historical biogeography

**Figure 4.**
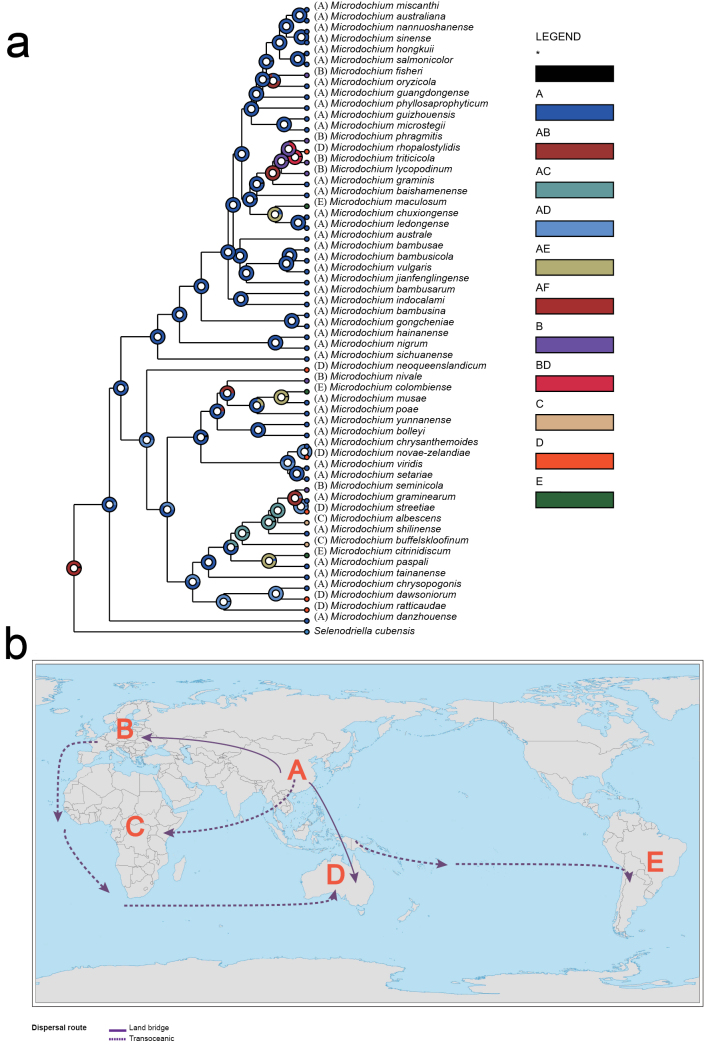
A ancestral state reconstruction and divergence time estimation of *Microdochium* using the ITS, LSU, *rpb2*, and *tub2* dataset. The pie chart in each node indicates the possible ancestral distributions inferred from Bayesian Binary Markov chain Monte Carlo analysis (BBM) implemented in RASP. A black asterisk represents other ancestral ranges. b Possible dispersal routes of *Microdochium*. Areas were marked as follows: (**A**) Asia, (**B**) Europe, (**C**) Africa, (**D**) Oceania, (**E**) South America.

The results of the inferred historical biogeographical scenarios regarding *Microdochium* using RASP are shown (Fig. 4). The results of Bayesian Binary Markov chain Monte Carlo (BBM) method analysis suggest a complex biogeographical history for *Microdochium*, with an origin most likely in Asia, and at least 17 dispersal events and 14 vicariance events determining the present distribution of the family *Microdochiaceae*. To date, 40 species have been found in Asia, 6 in Europe, 2 in Africa, 6 in Oceania and 3 in South America, suggesting that Asia is still the center of *Microdochium*.

### Taxonomy

#### Microdochium bambusicola

Taxon classificationFungiAmphisphaerialesAmphisphaeriaceae

Y.X. Shang, Q.Y. Liu, X.G. Zhang & Z. Li sp. nov.

5A99A10F-3998-5CB9-B2B9-ABEA7E6801B7

Fungal Names: FN 573512

Fig. 5

##### Etymology.

The specific epithet “bambusicola” refers to the host plant bamboo (*Poaceae* sp.).

**Figure 5.**
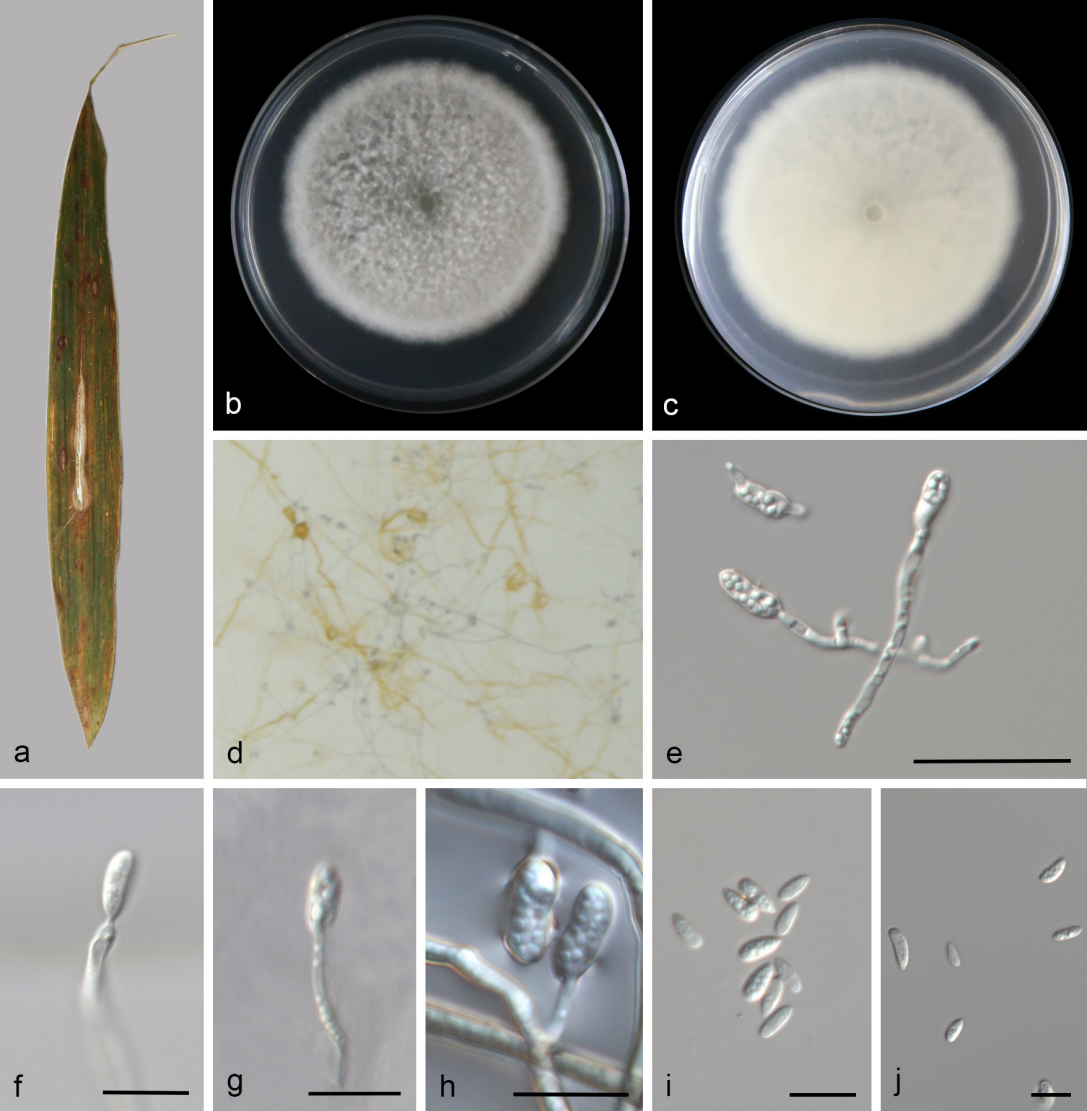
Morphology of *Microdochium bambusicola* (ex-type CGMCC 3.29432). **a** on leaf of bamboo (*Poaceae* sp.) **b, c** colonies (b-above, c-reverse) after 7 days on PDA**d** colony on PDA producing conidia masses **e–g** conidiophores with conidiogenous cells and conidia **h** conidiogenous cells and conidia **i–j** conidia. Scale bars: 10 μm (**e–j**).

##### Type.

CHINA • Hainan Province, Lingshui Li Autonomous County, Diaoluoshan, on leaves of bamboo (*Poaceae* sp.), 27 March 2023, Y.X. Shang (holotype HSAUP 7623-2), ex-type culture SAUCC 7623-2 = CGMCC 3.29432.

##### Description.

On leaves of bamboo (*Poaceae* sp.). Asexual morph: ***Mycelium*** immersed and superficial, abundant, hyphae hyaline, branched, septate, thin-walled, guttulate, 2.54–3.18 µm wide. ***Conidiophores*** solitary, simple, rarely branched, bent, cylindrical, 12.95–22.79 × 0.69–1.87 µm (av. = 16.83 ± 4.31 × 1.36 ± 0.59 μm, n = 14), often not clearly differentiated from the mycelium, hyaline. ***Conidiogenous cells*** terminal, monoblastic, cylindrical, 6.85–7.66 × 1.32–2.02 μm (av. = 7.21 ± 0.36 × 1.74 ± 0.33 μm, n = 11). ***Conidia*** dry, reniform, fusiform or cylindrical, smooth with thin walls, hyaline, round apex while the base is pointed, 6.28–11.12 × 2.85–4.11 μm (av. = 8.82 ± 1.55 × 3.41 ± 0.41 μm, n = 21), multi-guttulate and sometimes borne directly from the hyphae. ***Chlamydospores*** not observed. Sexual morphs unknown, see Fig. 5.

##### Culture characteristics.

Colonies on PDA. 66.49–67.67 mm in diameter after 7 d at 25 °C in darkness, with a growth rate of 9.50–9.67 mm/day, entire at edge, gray in obverse and cream-colored reverse.

##### Additional specimen examined.

CHINA • Hainan Province, Lingshui Li Autonomous County, Diaoluoshan, on leaves of bamboo (*Poaceae* sp.), 27 March 2023, Y.X. Shang (HSAUP 7623-2A), living culture SAUCC 7623-2A.

##### Notes.

*Microdochium bambusicola* belongs to the large clade, where it shows a relationship with *M. bambusae* (SAUCC 1862-1) (Fig. 1). In phylogeny, *M. bambusicola* differs from *M. bambusae* by 1 nucleotides in ITS, 4 nucleotides in LSU, 30 nucleotides in *rpb2*, and 14 nucleotides in *tub2*. In morphology, *M. bambusicola* is distinguished from *M. bambusae* by the size of conidiogenous cells (6.85–7.66 × 1.32–2.02 μm vs. 17.4–30.0 × 2.5–3.0 µm) and conidia (6.28–11.12 × 2.85–4.11 μm vs. 13.0–17.0 × 2.5–3.5 µm) (Zhang et al. 2023a). Therefore, based on morphology and phylogenetic evidence, we establish fungus as *Microdochium bambusicola* sp. nov.

#### Microdochium

Taxon classificationFungiAmphisphaerialesAmphisphaeriaceae

Syd., Ann. Mycol. 22: 267. 1924

8ED6FABD-107E-5153-BEA9-5E06E164C53E

= Monographella Petr., Ann. Mycol. 22: 144. 1924.
= Griphosphaerella Petr., Ann. Mycol. 25: 209. 1927.
= Gloeocercospora D.C. Bain & Edgerton, Trans. Brit. Mycol. Soc. 57: 358. 1971.
= Gerlachia W. Gams & E. Müll., Netherlands J. Agric. Sci. 86: 49. 1980.
= Macroidriella Z.X. Zhang, J.W. Xia & X.G. Zhang, MycoKeys. 106: 303–325. 2024.

##### Type species.

*Microdochium phragmitis* Syd.

##### Description.

Based on Hernández-Restrepo et al. (2016) and our own observations, the asexual morph of the genus ***Microdochium*** is characterized as follows. The ***mycelium*** is immersed, branched, and septate, with hyphae ranging from hyaline to pale brown. ***Sporodochia***, when present, are located within or beneath the host epidermis. They emerge through stomata, by rupturing the outer epidermal wall and cuticle, or via specialized egression hyphae that penetrate the outer epidermal wall. The sporodochia are hyaline, composed of pseudoparenchyma, and expand after emerging from the host tissue. ***Conidiophores*** are more or less verticillate and often reduced to conidiogenous cells. ***Conidiogenous cells*** are holoblastic and discrete, occurring singly or aggregated in small sporodochia; they may exhibit sympodial proliferation (cylindrical to clavate, with apical denticles) or percurrent/annellidic proliferation (subcylindrical to lageniform). ***Conidia*** are dry, or in slimy masses, unicellular to multiseptate, hyaline and smooth; their shapes include lunate, falcate, fusiform, filiform, obovoid or subpyriform, straight or curved, with rounded apex and flattened base. ***Chlamydospores*** are terminal or intercalary, brown, and may be solitary, in chains, or clustered. The sexual morph is ***Monographella***-like, characterized by perithecial ***ascomata*** with a papillate ostiole, unitunicate asci with an amyloid apical ring, and clavate to fusoid ascospores (see Hernández-Restrepo et al. 2016).

##### Notes.

The genus *Microdochium* was introduced with *M. phragmitis* as its type species, isolated from *Phragmites australis* in Germany. The genus is characterized by hyaline conidiogenous cells and conidia that are highly variable in shape, ranging from cylindrical, fusoid, or oblong to lunate, straight or curved, with truncate bases and apices rounded (Sydow 1924; Hernández-Restrepo et al. 2016). Recently, Zhang et al. (2024) proposed the genus *Macroidriella*, typified by *Macroidriella bambusae*, based on its lunate and curved conidia. However, the conidial morphology of *Microdochium* is known to include lunate and curved forms (Sydow 1924; Hernández-Restrepo et al. 2016). Therefore, the lunate and curved conidia alone do not justify the establishment of *Macroidriella* as a distinct genus. Based on phylogenetic analyses, divergence time estimation, and morphological comparisons, we formally propose to synonymize *Macroidriella* Z.X. Zhang, J.W. Xia & X.G. Zhang under *Microdochium* Syd. Consequently, the type species *Macroidriella bambusae* is transferred to *Microdochium* as a new name below.

#### Microdochium danzhouense

Taxon classificationFungiAmphisphaerialesAmphisphaeriaceae

(Z.X. Zhang & X.G. Zhang) Q.Y. Liu & X.G. Zhang nom. nov.

1062A663-0466-52E5-9220-9CC59D26BE01

Fungal Names: FN 573520

##### Etymology.

The epithet “danzhouense” denotes the geographical origin of the strains, namely Danzhou City.

##### Basionym.

*Macroidriella bambusae* Z.X. Zhang & X.G. Zhang.

##### Description.

See Zhang et al. (2024).

##### Distribution.

China.

##### Ecology.

Associated with leaf diseases of *Bambusaceae* sp. and dead leaves.

##### Notes.

Five isolates obtained from diseased leaves of *Bambusaceae* sp. and dead leaves formed an independent clade, which is newly described here as *M. danzhouense*. This species is phylogenetically closely related to *M. neoqueenslandicum* (Fig. 1). In phylogeny, *M. danzhouense* differs from *M. neoqueenslandicum* by 246 nucleotides (48/533 in ITS, 14/836 in LSU, 36/629 in *tub2*, 148/841 in *rpb2*). In morphology, *M. danzhouense* is distinguished from *M. neoqueenslandicum* by the conidial size (16.5–21.7 × 2–2.8 µm vs. 4–9 × 1.5–3 μm) and conidiogenous cell length (10.4–15 µm vs. 4.5–10 μm) (Hernández-Restrepo et al. 2016; Zhang et al. 2024).

#### Microdochium guangdongense

Taxon classificationFungiAmphisphaerialesAmphisphaeriaceae

Y.X. Shang, Q.Y. Liu, X.G. Zhang & Z. Li sp. nov.

E7CDF6F2-F0D6-56E5-9F26-60E6FABD19C1

Fungal Names: FN 573513

Fig. 6

##### Etymology.

The epithet “guangdongense” refers to the location where the type was collected Guangdong Province.

**Figure 6.**
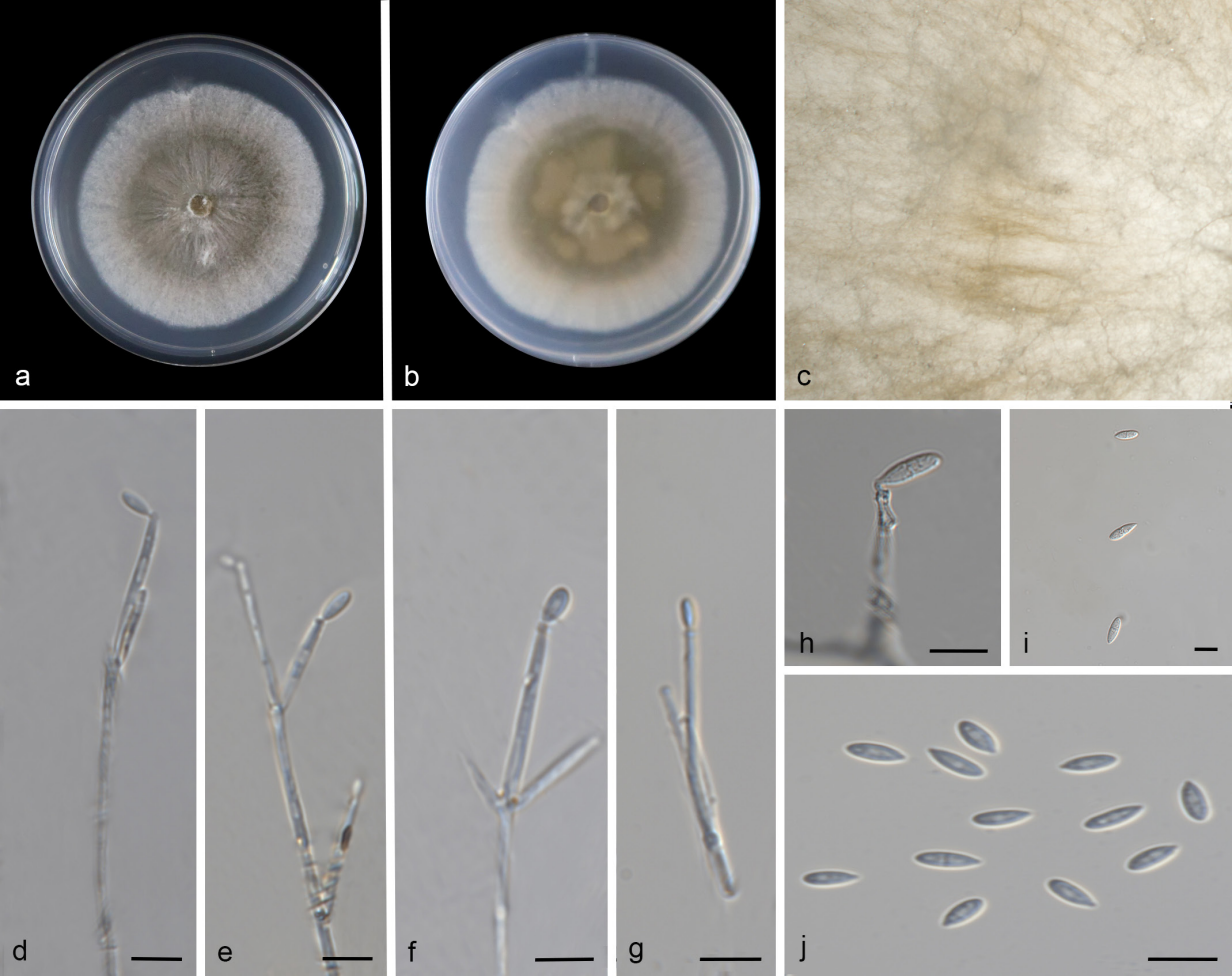
Morphology of *Microdochium guangdongense* (ex-type CGMCC 3.29429). **a, b** colonies (a-above, b-reverse) after 7 d on PDA**c** colony overview **d–h** conidiophores with conidiogenous cells and conidia **i, j** conidia. Scale bars: 10 μm (**d–j**).

##### Type.

CHINA • Guangdong Province, Guangzhou City, Conghua District, on leaves of *Musa basjoo*, 01 March 2025, Y.X. Shang (holotype HSAUP 17239-1), ex-type culture SAUCC 17239-1 = CGMCC 3.29429.

##### Description.

On leaves of *Musa basjoo*. Asexual morph: ***Mycelium*** immersed and superficial, abundant, hyphae hyaline, branched, septate, thin-walled, guttulate, 2.44–4.32 µm wide. ***Conidiophores*** solitary, simple, branched, bent, cylindrical, 62.42–90.03 × 2.23–3.07 µm, often not clearly differentiated from the mycelium, hyaline. ***Conidiogenous cells*** terminal or lateral, monoblastic, cylindrical, 19.86–45.26 × 1.73–3.69 μm (av. = 30.19 ± 7.95 × 2.49 ± 0.59 μm, n = 17). ***Conidia*** dry, fusiform, smooth with thin walls, hyaline, round apex while the base is pointed, (0–1)-septate, 7.64–11.09 × 3.03–4.64 μm (av. = 9.32 ± 0.82 × 3.74 ± 0.37 μm, n = 20). ***Chlamydospores*** not observed. Sexual morphs unknown, see Fig. 6.

##### Culture characteristics.

Colonies on PDA. 68.44–73.01 mm in diameter after 7 d at 25 °C in darkness, with a growth rate of 9.78–10.43 mm/day, entire at edge, tanned in the center, white at the margin in obverse and reverse.

##### Additional specimen examined.

CHINA • Guangdong Province, Guangzhou City, Conghua District, on leaves of *Musa basjoo*, 01 March 2025, Y.X. Shang (HSAUP 17239-1A), living culture SAUCC 17239-1A.

##### Notes.

*Microdochium guangdongense* belongs to the large clade, where it shows a relationship with *M. fisheri* (CBS 242.90) (Fig. 1). In phylogeny, *M. guangdongense* differs from *M. fisheri* by 126 nucleotides (9/527 in ITS, 5/835 in LSU, 70/740 in rpb2, 42/684 in *tub2*). In morphology, the conidiophores of the *M. fisheri* are characteristically bifurcate, while those of the *M. guangdongense* are simple or irregularly branched and often bent. *M. guangdongense* is distinguished from *M. fisheri* by the conidiogenous cells (19.86–45.26 × 1.73–3.69 μm vs. 19–60 × 1.5–2 μm) (Hernández-Restrepo et al. 2016). Therefore, based on morphology and phylogenetic evidence, we establish fungus as *Microdochium guangdongense* sp. nov.

#### Microdochium guizhouensis

Taxon classificationFungiAmphisphaerialesAmphisphaeriaceae

Norph. & S.Q. Guo, MYCOSPHERE 16 (2): 1–78 (2025)

9680BD65-78B3-5FE7-94D4-C9C3B2E5236A

Fig. 7

##### Description.

**Figure 7.**
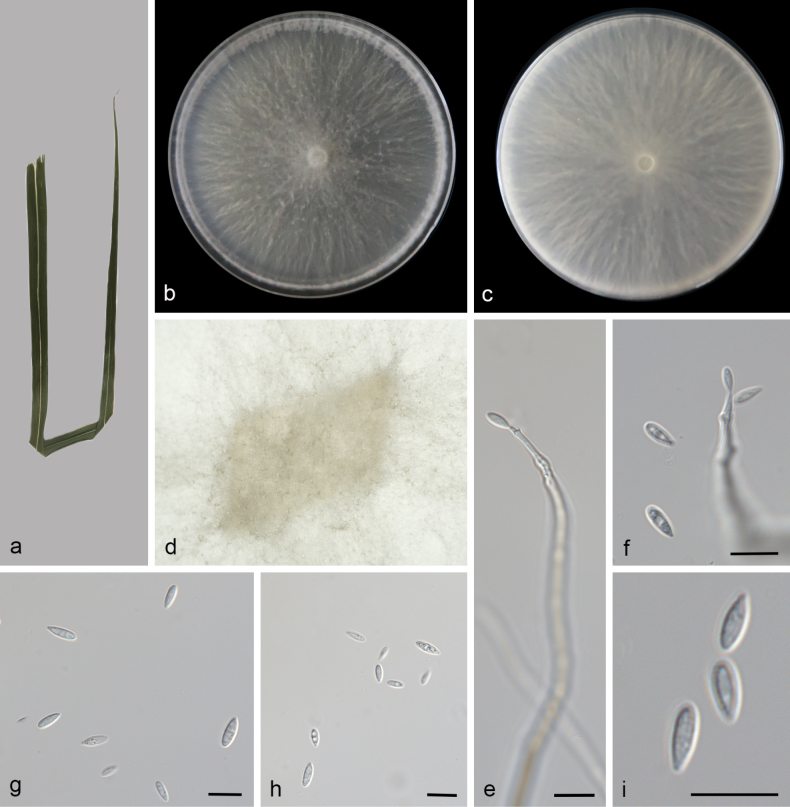
Morphology of *Microdochium guizhouensis* (SAUCC 11159-1). **a** on leaf of *Phragmites australis***b, c** colonies (b-above, c-reverse) after 7 d on PDA**d** colony on PDA producing conidia masses **e, f** conidiophores with conidiogenous cells and conidia **g–i** conidia. Scale bars: 10 μm (**e–i**).

On leaves of *Phragmites australis*. Asexual morph: ***Mycelium*** immersed and superficial, moderate, hyphae hyaline, branched, septate, thin-walled, 1.56–2.44 µm wide. ***Conidiomata*** sporodochial, giving rise to pale orange conidial mass. ***Conidiophores*** solitary, acrogenous, simple, rarely branched, straight, rarely bent, cylindrical, 30.62–92.43 × 2.23–3.58 µm, often not clearly differentiated from the mycelium, hyaline to pale brown. ***Conidiogenous cells*** terminal, monoblastic, cylindrical, 11.26–12.77 × 1.65–1.48 μm. ***Conidia*** dry, fusiform, straight, smooth with thin walls, hyaline, (0–1)-septate, 5.23–9.43 × 2.19–3.14 μm (av. = 7.51 ± 1.17 × 2.63 ± 0.29 μm, n = 23), round apex while the base is pointed and tapers to a distinct scar. ***Chlamydospores*** not observed. Sexual morphs unknown, see Fig. 7.

##### Culture characteristics.

Colonies on PDA. 90 mm in diameter after 7 d at 25 °C in darkness, with a growth rate of 12.86 mm/day, entire at edge, white in obverse and reverse.

##### Materials examined.

CHINA • Fujian Province, Nanping City, West of the Longguiyuan Ticket Office, on leaves of *Phragmites australis*, 20 October 2024, Y.X. Shang (HSAUP 11159-1), living culture SAUCC 11159-1.

##### Distribution.

China, Fujian and Guizhou Province.

##### Ecology.

Associated with diseased leaves of *Phragmites australis* and cabbage root soil.

##### Notes.

This isolate obtained from leaves of *Phragmites australis* formed a well-supported clade with two isolates of *Microdochium guizhouensis* from soil in Chinese cabbage root (Fig. 1; Hongsanan et al. 2025). Hence, *Phragmites australis* become a new host for *M. guizhouensis*.

#### Microdochium ledongense

Taxon classificationFungiAmphisphaerialesAmphisphaeriaceae

Y.X. Shang, Q.Y. Liu, X.G. Zhang & Z. Li sp. nov.

C27A2928-E211-5F2B-923C-B258729EFD5B

Fungal Names: FN 573514

Fig. 8

##### Etymology.

The epithet “ledongense” refers to the location where the type was collected, Ledong Li Autonomous County.

**Figure 8.**
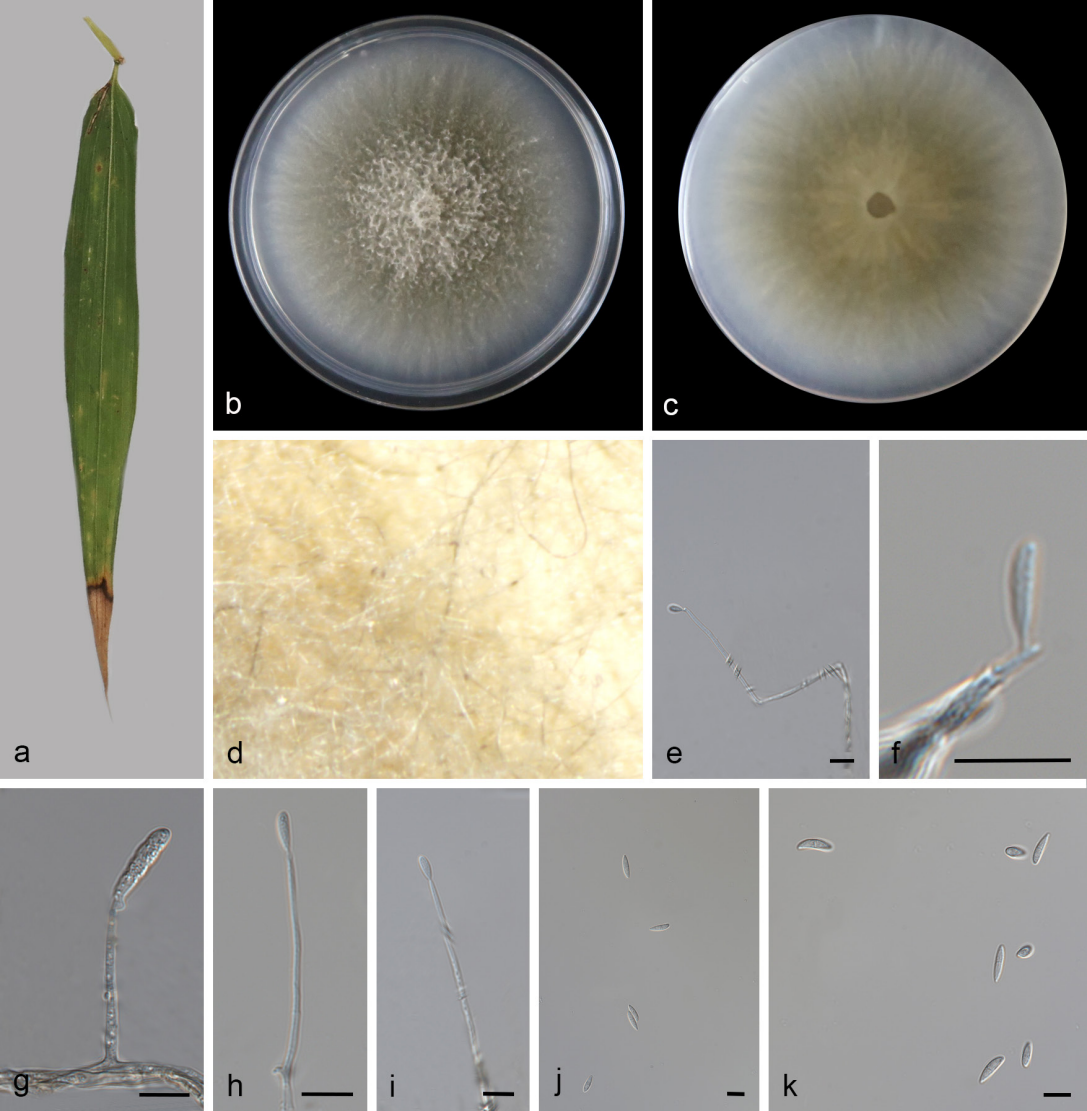
Morphology of *Microdochium ledongense* (ex-type CGMCC 3.29650). **a** on leaf of bamboo (*Poaceae* sp.) **b, c** colonies (b-above, c-reverse) after 7 d on PDA**d** colony overview **e–i** conidiophores with conidiogenous cells and conidia **j, k** conidia. Scale bars: 10 μm (**e–k**).

##### Type.

CHINA • Hainan Province, Ledong Li Autonomous County, Jianfengling National Forest Park, on leaves of bamboo (*Poaceae* sp.), 04 December 2024, Y.X. Shang (holotype HSAUP 14499-3), ex-type culture SAUCC 14499-3 = CGMCC 3.29650.

##### Description.

On leaves of bamboo (*Poaceae* sp.). Asexual morph: ***Mycelium*** immersed and superficial, abundant, hyphae hyaline to brown, branched, septate, thin-walled, guttulate, 1.58–2.56 µm wide. ***Conidiophores*** solitary, simple, rarely branched, bent, cylindrical, 49.72–71.04 × 1.47–2.48 µm, often not clearly differentiated from the mycelium. ***Conidiogenous cells*** terminal, monoblastic, cylindrical, 31.63 × 3.38 μm. ***Conidia*** dry, fusoid or cylindrical, (0–1)-septate, smooth with thin walls, hyaline, round apex while the base is pointed, 10.03–13.41 × 2.24–3.78 μm (av. = 11.91 ± 1.10 × 3.14 ± 0.46 μm, n = 23), sometimes borne directly from the hyphae. ***Chlamydospores*** not observed. Sexual morphs unknown, see Fig. 8.

##### Culture characteristics.

Colonies on PDA. 90 mm in diameter after 7 d at 25 °C in darkness, with a growth rate of 12.86 mm/day, entire at edge, medium brown in obverse and reverse.

##### Additional specimen examined.

CHINA • Hainan Province, Lingshui Li Autonomous County, Diaoluoshan, on leaves of bamboo (*Poaceae* sp.), 06 March 2024, Y.X. Shang (HSAUP 16723-1), living culture SAUCC 16723-1; • ibid. HSAUP 17376-5, living culture SAUCC 17376-5; • ibid. HSAUP 16889-3, living culture SAUCC 16889-3; • ibid. HSAUP 16889-1, living culture SAUCC 16889-1; • ibid. on leaves of *Sasaella masamuneana*, 06 March 2025, Y.X. Shang (HSAUP 16957-1), living culture SAUCC 16957-1.

##### Notes.

*Microdochium ledongense* belongs to the large clade, where it shows a relationship with *M. chuxiongense* (YFCC 8794) (Fig. 1). In phylogeny, *M. ledongense* differs from *M. chuxiongense* by 28 nucleotides (3/427 in ITS, 6/549 in LSU, 12/842 in *rpb2*, and 7/670 in *tub2*). In morphology, *M. ledongense* is distinguished from *M. chuxiongense* by its conidiophores length (49.72–71.04 × 1.47–2.48 µm vs. 8–15 × 2–3 μm) and conidia size (10.03–13.41 × 2.24–3.78 μm vs. 4–12 × 2–5 μm) (Tang et al. 2022). Therefore, based on morphology and phylogenetic evidence, we establish fungus as *Microdochium ledongense* sp. nov.

#### Microdochium microstegii

Taxon classificationFungiAmphisphaerialesAmphisphaeriaceae

Y.X. Shang, Q.Y. Liu, X.G. Zhang & Z. Li sp. nov.

655CD778-5642-5B4E-8355-001413CD43BE

Fungal Names: FN 573515

Fig. 9

##### Etymology.

The specific epithet “microstegii” refers to the host plant *Microstegium vimineum*.

**Figure 9.**
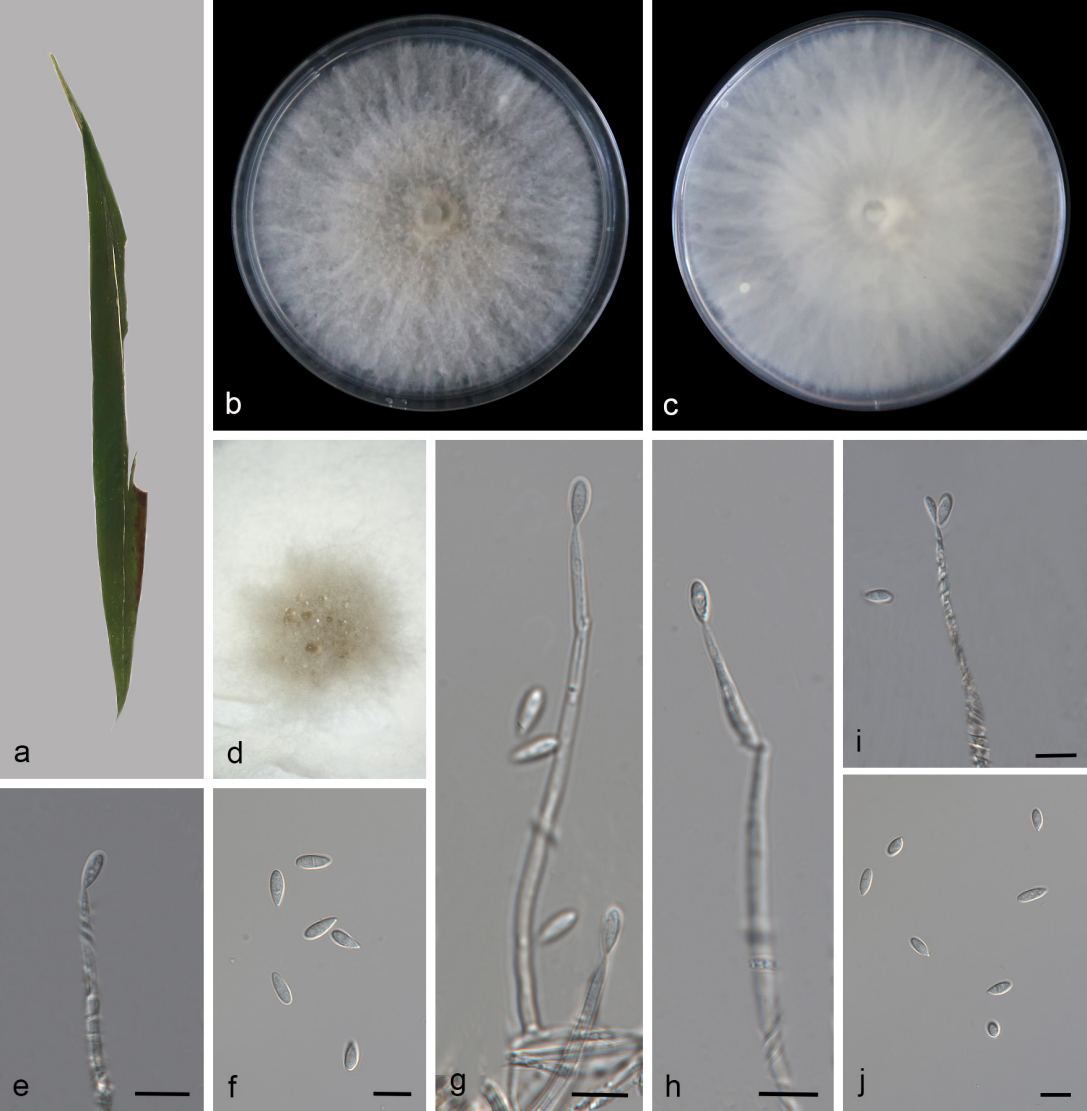
Morphology of *Microdochium microstegii* (ex-type CGMCC 3.29421). **a** on leaf of *Microstegium vimineum***b, c** colonies (b-above, c-reverse) after 7 d on PDA**d** Colony on PDA producing conidia masses **e, g–i** conidiophores with conidiogenous cells and conidia **f, j** conidia. Scale bars: 10 μm (**e–j**).

##### Type.

CHINA • Hainan Province, Qiongzhong Li and Miao Autonomous County, on leaves of *Setaria viridis*, 15 April 2025, Y.X. Shang (holotype HSAUP 14288-1), ex-type culture SAUCC 14288-1 = CGMCC 3.29421.

##### Description.

On leaves of *Microstegium vimineum*. Asexual morph: ***Mycelium*** immersed and superficial, abundant, hyphae hyaline, branched, septate, thin-walled, 1.60–2.21 µm wide. ***Conidiophores*** solitary, acrogenous, simple, rarely branched, straight, rarely bent, cylindrical, 39.27–93.39 × 2.35–3.04 µm, often not clearly differentiated from the mycelium, hyaline to pale brown. ***Conidiogenous cells*** terminal, monoblastic or polyblastic, cylindrical, 18.07–30.30 × 2.44–3.67 μm. ***Conidia*** dry, fusiform, straight, smooth with thin walls, hyaline, (0–1)-septate, 5.55–9.84 × 2.53–4.17 μm (av. = 7.51 ± 1.19 × 3.19 ± 0.62 μm, n = 23). ***Chlamydospores*** not observed. Sexual morphs unknown, see Fig. 9.

##### Culture characteristics.

Colonies on PDA. 90 mm in diameter after 7 d at 25 °C in darkness, with a growth rate of 12.86 mm/day, entire at edge, white in obverse and reverse.

##### Additional specimen examined.

CHINA • Hainan Province, Wuzhishan City, Hong Canyon Scenic Area, on leaves of *Microstegium vimineum*, 15 December 2025, Y.X. Shang (HSAUP 14103-3), living culture SAUCC 14103-3. CHINA • Guangxi Zhuang Autonomous Region, Liuzhou City, Guting Mountain Forest Park, on leaves of *Setaria viridis*, 15 April 2025, Y.X. Shang (HSAUP 18044-6), living culture SAUCC 18044-6.

##### Notes.

*Microdochium microstegii* belongs to the large clade, where it shows a relationship with *M. guizhouensis* (GUCC 25–0012) (Fig. 1). In phylogeny, *M. microstegii* differs from *M. guizhouensis* by 40 nucleotides in *rpb2* and 19 nucleotides in *tub2*. In morphology, *M. microstegii* is distinguished from *M. guizhouensis* by the length of conidiogenous cells (18.07–30.30 μm vs. 15–17(–40) µm) and conidiophores (39.27–93.39 µm vs. 13–40(–150) µm), Hongsanan et al. (2025) described the conidiophores of *M. guizhouensis* as 40–13(–150) × 2.5–3.5 µm, but my measurements from their figures indicate they are 13–40(–150) × 2.5–3.5 µm. Therefore, based on morphology and phylogenetic evidence, we establish fungus as *Microdochium microstegii* sp. nov.

#### Microdochium miscanthi

Taxon classificationFungiAmphisphaerialesAmphisphaeriaceae

S.B. Liu, X.Y. Liu, Z. Meng & X.G. Zhang, Journal of Fungi 8 (6), 577 (2022)

B8A581E9-3E42-5144-862F-3ADC52449DB7

Fig. 10

##### Description.

**Figure 10.**
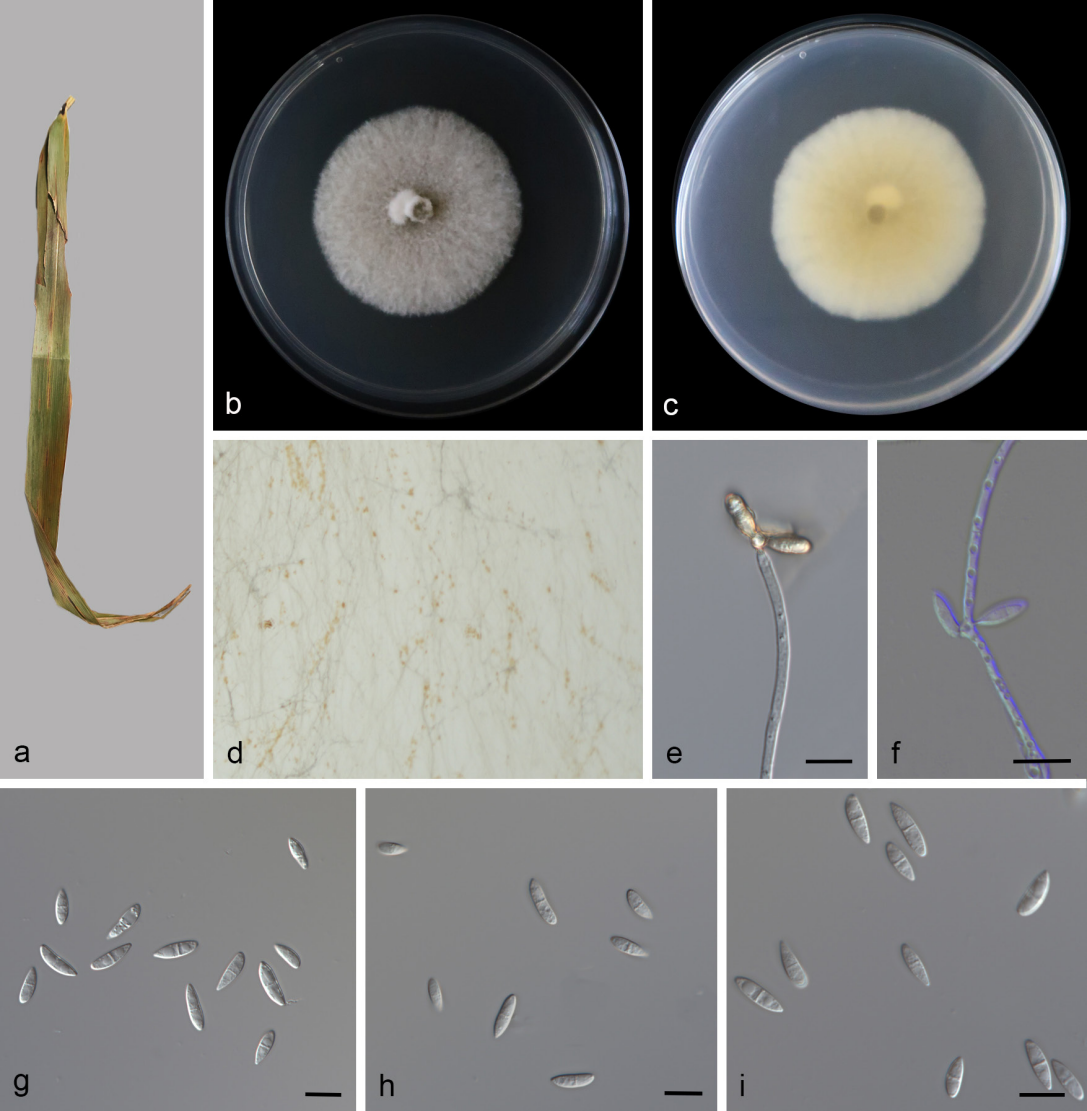
Morphology of *Microdochium miscanthi* (SAUCC 7610-2). **a** on leaf of *Phragmites australis***b, c** colonies (b-above, c-reverse) after 7 d on PDA**d** colony on PDA producing conidia masses **e** conidiophores with conidiogenous cells and conidia **f** hyaline, conidiogenous cells and conidia **g–i** conidia. Scale bars: 10 μm (**e–i**).

On leaves of *Phragmites australis*. Asexual morph: ***Mycelium*** superficial, abundant, hyphae hyaline, branched, septate, thin-walled, 1.99–3.04 µm wide. ***Conidiophores*** solitary, simple, rarely branched, straight, rarely bent, cylindrical, 49.70 × 2.96 µm, often not clearly differentiated from the mycelium, hyaline. ***Conidiogenous cells*** terminal or lateral, monoblastic or polyblastic, cylindrical, 2.21–3.44 × 2.21–2.87 μm. ***Conidia*** dry, fusiform, straight, smooth with thin walls, hyaline, (0–1)-septate, 9.21–14.07 × 2.96–4.39 μm (av. = 11.54 ± 1.31 × 3.50 ± 0.48 μm, n = 26), sometimes borne directly from hyphae. ***Chlamydospores*** not observed. Sexual morphs unknown, see Fig. 10.

##### Culture characteristics.

Colonies on PDA. 48.45–50.35 mm in diameter after 7 d at 25 °C in darkness, with a growth rate of 6.92–7.19 mm/day, entire at edge, white in obverse and pale yellow reverse.

##### Materials examined.

CHINA • Hainan Province, Lingshui Li Autonomous County, Diaoluoshan, on leaves of *Phragmites australis*, 27 March 2023, Y.X. Shang (HSAUP 7610-2), living culture SAUCC 7610-2; • ibid. on leaves of *Phyllostachys nigra*, 27 March 2023, Y.X. Shang (HSAUP 7638-2A), living culture SAUCC 7638-2A; • ibid. on leaves of *Pseudosasa japonica*, 30 November 2024, Y.X. Shang (HSAUP 13968-2), living culture SAUCC 13968-2; • ibid. Wuzhishan City, Hongxiagu Scenic Area, on leaves of *Licuala grandis*, 01 December 2024, Y.X. Shang (HSAUP 14090-2), living culture SAUCC 14090-2.

##### Distribution.

China, Hainan Province.

##### Ecology.

Associated with diseased leaves of *Licuala grandis*, *Miscanthus sinensis*, *Phragmites australis*, *Phyllostachys nigra*, and *Pseudosasa japonica*.

##### Notes.

These isolates obtained from leaves of *Licuala grandis*, *Phragmites australis*, *Phyllostachys nigra*, and *Pseudosasa japonica* formed a well-supported clade with two isolates of *Microdochium miscanthi* from *Miscanthus sinensis* leaves (Fig. 1; Liu et al. 2022). Hence, *Phragmites australis*, *Phyllostachys nigra*, *Pseudosasa japonica*, and *Licuala grandis* become new hosts for *M. miscanthi*.

#### Microdochium nannuoshanense

Taxon classificationFungiAmphisphaerialesAmphisphaeriaceae

J. Zhang, Z.X. Zhang, & Z. Li, Journal of Fungi 9, 1176 (2023)

3D6939BE-104B-50ED-B3D4-728A73237600

Fig. 11

##### Description.

**Figure 11.**
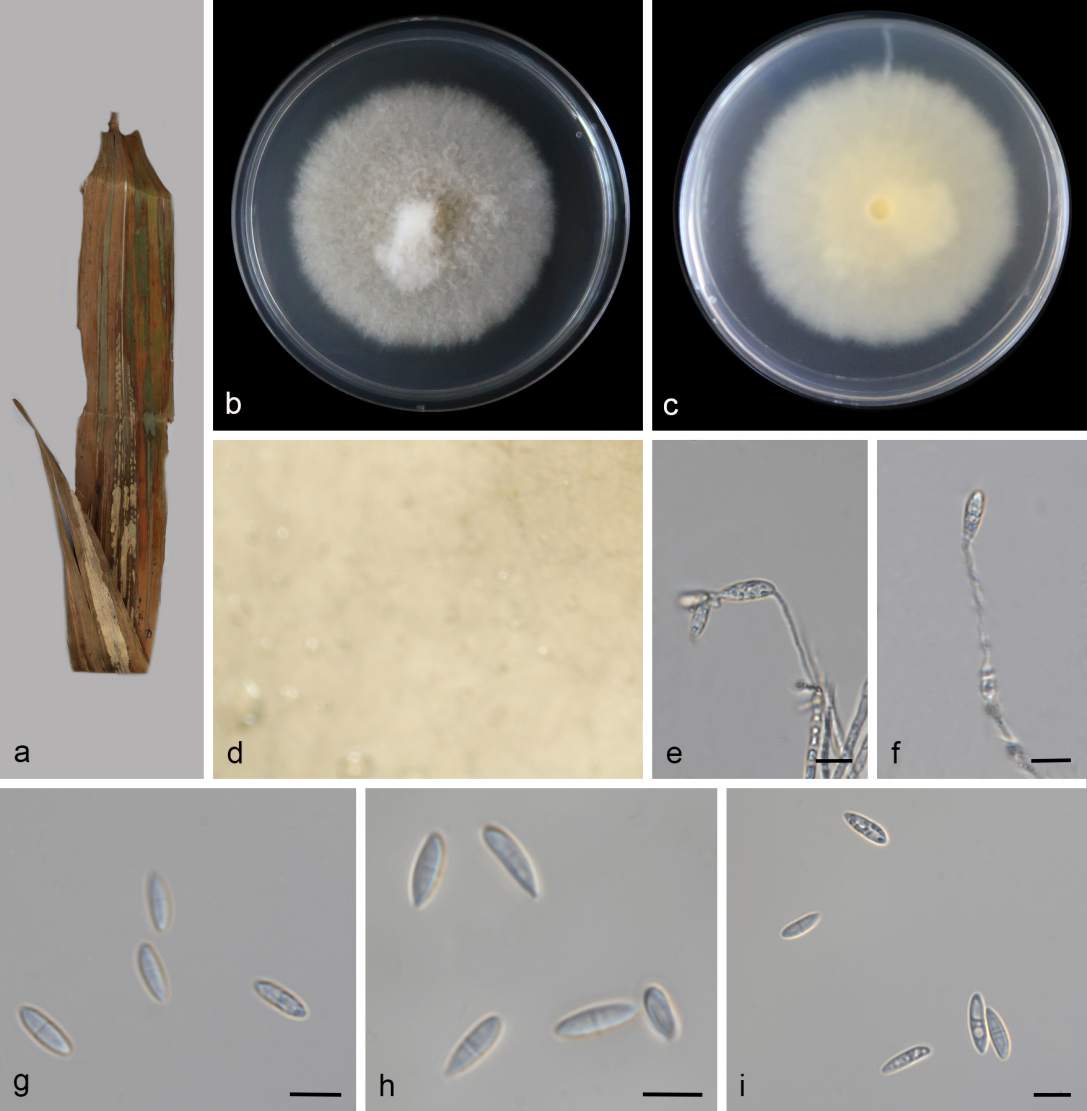
Morphology of *Microdochium nannuoshanense* (SAUCC 13988-3). **a** on unknown diseased leaf **b, c** colonies (b-above, c-reverse) after 7 d on PDA**d** conidiomata formed on PDA**e**, **f** conidiophores with conidiogenous cells and conidia **g–i** conidia. Scale bars: 10 μm (**e–i**).

Asexual morph: ***Mycelium*** superficial, abundant, hyphae hyaline, branched, septate, thin-walled, 1.73–2.92 µm wide. ***Conidiophores*** solitary, simple, rarely branched, bent, cylindrical, 49.70 × 2.96 µm, often not clearly differentiated from the mycelium, hyaline. ***Conidiogenous cells*** terminal, monoblastic or polyblastic, cylindrical, 17.04–38.92 × 2.08–2.10 μm. ***Conidia*** dry, fusiform, straight, smooth with thin walls, hyaline, (0–)1-septate, 9.47–15.99 × 2.86–5.30 μm (av. = 12.51 ± 1.95 × 3.93 ± 0.66 μm, n = 23). ***Chlamydospores*** not observed. Sexual morphs unknown, see Fig. 11.

##### Culture characteristics.

Colonies on PDA. 63.03–66.96 mm in diameter after 7 d at 25 °C in darkness, with a growth rate of 9.00–9.57 mm/day, entire at edge, grayish in obverse and pale yellow reverse.

##### Materials examined.

CHINA • Hainan Province, Lingshui Li Autonomous County, Country Road 124, on unknown diseased leaves, 30 November 2024, Y.X. Shang (HSAUP 13988-3), living culture SAUCC 13988-3.

##### Distribution.

China, Hainan and Yunnan Province.

##### Ecology.

Associated with unknown diseased leaves and on diseased leaves of *Bambusaceae* sp.

##### Notes.

This isolate obtained from unknown diseased leaves formed a well-supported clade with two isolates of *Microdochium nannuoshanense* from Yunnan (Fig. 1; Zhang et al. 2023a). Hence, Hainan became a new geographical record for *M. nannuoshanense*.

#### Microdochium nigrum

Taxon classificationFungiAmphisphaerialesAmphisphaeriaceae

Y.X. Shang, Q.Y. Liu, X.G. Zhang & Z. Li sp. nov.

D9EA6BF6-FA38-5522-9976-3EEEEFC4DE57

Fungal Names: FN 573516

Fig. 12

##### Etymology.

The specific epithet “nigrum” refers to the host plant *Phyllostachys nigra*.

**Figure 12.**
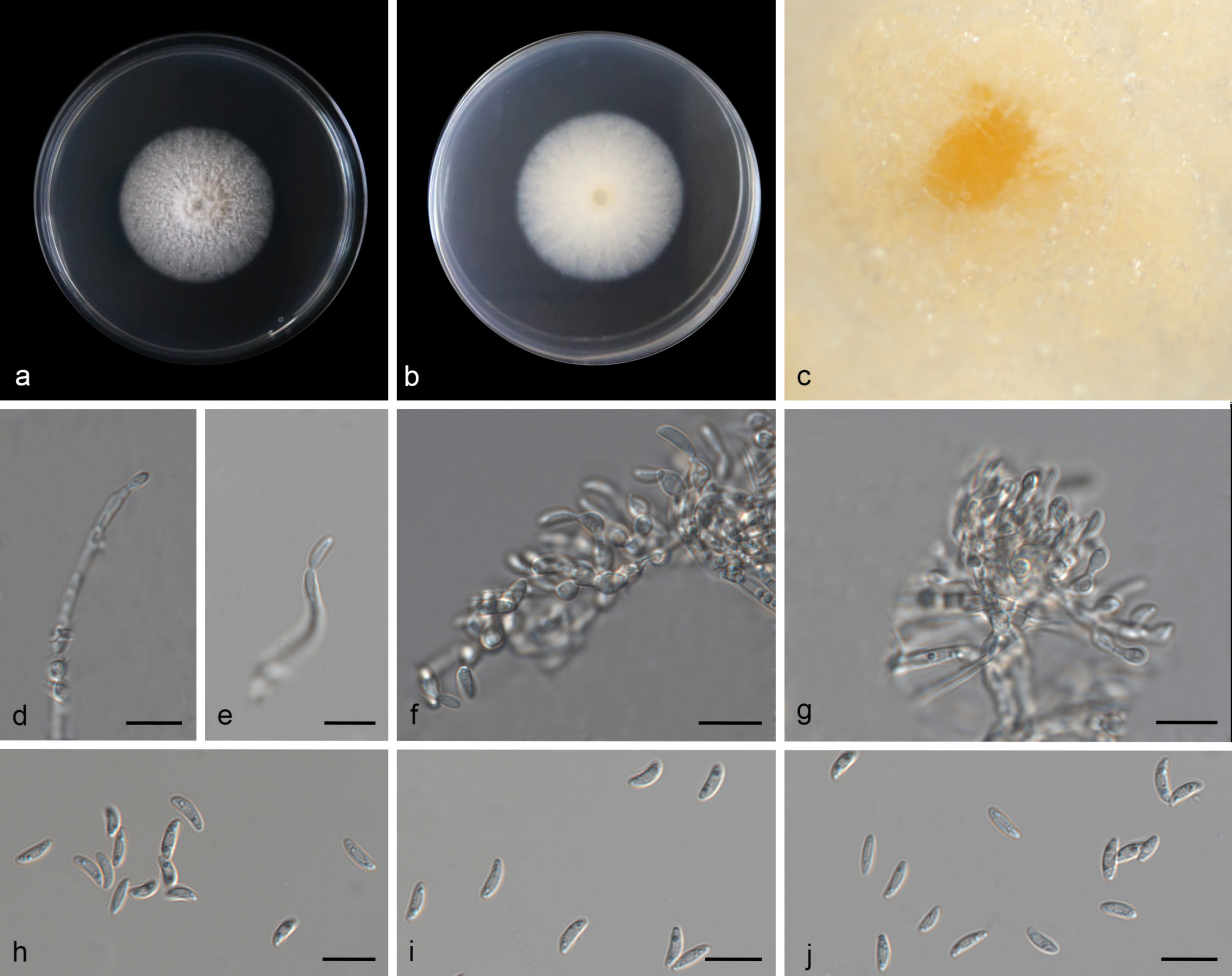
Morphology of *Microdochium nigrum* (ex-type CGMCC 3.29428). **a, b** colonies (a-above, b-reverse) after 7 d on PDA**c** colony on PDA producing conidia masses **d–g** conidiophores with conidiogenous cells and conidia **h–j** conidia. Scale bars: 10 μm (**d–j**).

##### Type.

CHINA • Guangdong Province, Shaoguan City, Renhua County, on leaves of *Phyllostachys nigra*, 03 March 2025, Y.X. Shang (holotype HSAUP 17349-2), ex-type culture SAUCC 17349-2 = CGMCC 3.29428.

##### Description.

On leaves of *Phyllostachys nigra*. Asexual morph: ***Mycelium*** immersed and superficial, abundant, hyphae hyaline, branched, septate, thin-walled, guttulate, 1.11–3.05 µm wide. ***Conidiophores*** more or less mono- or biverticillate, metulae clavate to cylindrical. ***Conidiogenous cells*** polyblastic, with percurrent proliferations, ampulliform, lageniform to subcylindrical, 4.47–10.25 × 1.90–3.42 μm (av. = 6.02 ± 2.03 × 2.40 ± 0.53 μm, n = 17). ***Conidia*** lunate, allantoid, curved, with one side straighter than the other, 5.92–8.61 × 1.72–2.69 μm (av. = 7.32 ± 0.71 × 2.22 ± 0.32 μm, n = 31), base flattened. Sometimes produced directly on the mycelial hyphae. ***Chlamydospores*** not observed. Sexual morphs unknown, see Fig. 12.

##### Culture characteristics.

Colonies on PDA. 45.21–47.19 mm in diameter after 7 d at 25 °C in darkness, with a growth rate of 6.45–6.74 mm/day, entire at edge, white in obverse and reverse.

##### Additional specimen examined.

CHINA • Guangdong Province, Shaoguan City, Renhua County, on leaves of *Phyllostachys nigra*, 03 March 2025, Y.X. Shang (HSAUP 17349-2A), living culture SAUCC 17349-2A.

##### Notes.

*Microdochium nigrum* belongs to the large clade, where it shows a relationship with *M. hainanense* (SAUCC 210781) (Fig. 1). In phylogeny, *M. nigrum* differs from *M. hainanense* by 91 nucleotides (9/534 in ITS, 54/841 in *rpb2*, and 28/ 693 in *tub2*). In morphology, *M. nigrum* is distinguished from *M. hainanense* by its conidia size (5.92–8.61 × 1.72–2.69 μm vs. 7.0–16.1 × 2.5–4.7 µm) (Liu et al. 2022). Therefore, based on morphology and phylogenetic evidence, we establish fungus as *Microdochium nigrum* sp. nov.

#### Microdochium setariae

Taxon classificationFungiAmphisphaerialesAmphisphaeriaceae

Y.X. Shang, Q.Y. Liu, X.G. Zhang & Z. Li sp. nov.

86F23BBB-9775-5B7F-BD0D-655199875BCF

Fungal Names: FN 573517

Fig. 13

##### Etymology.

The specific epithet “setariae” refers to the host plant *Setaria viridis*.

**Figure 13.**
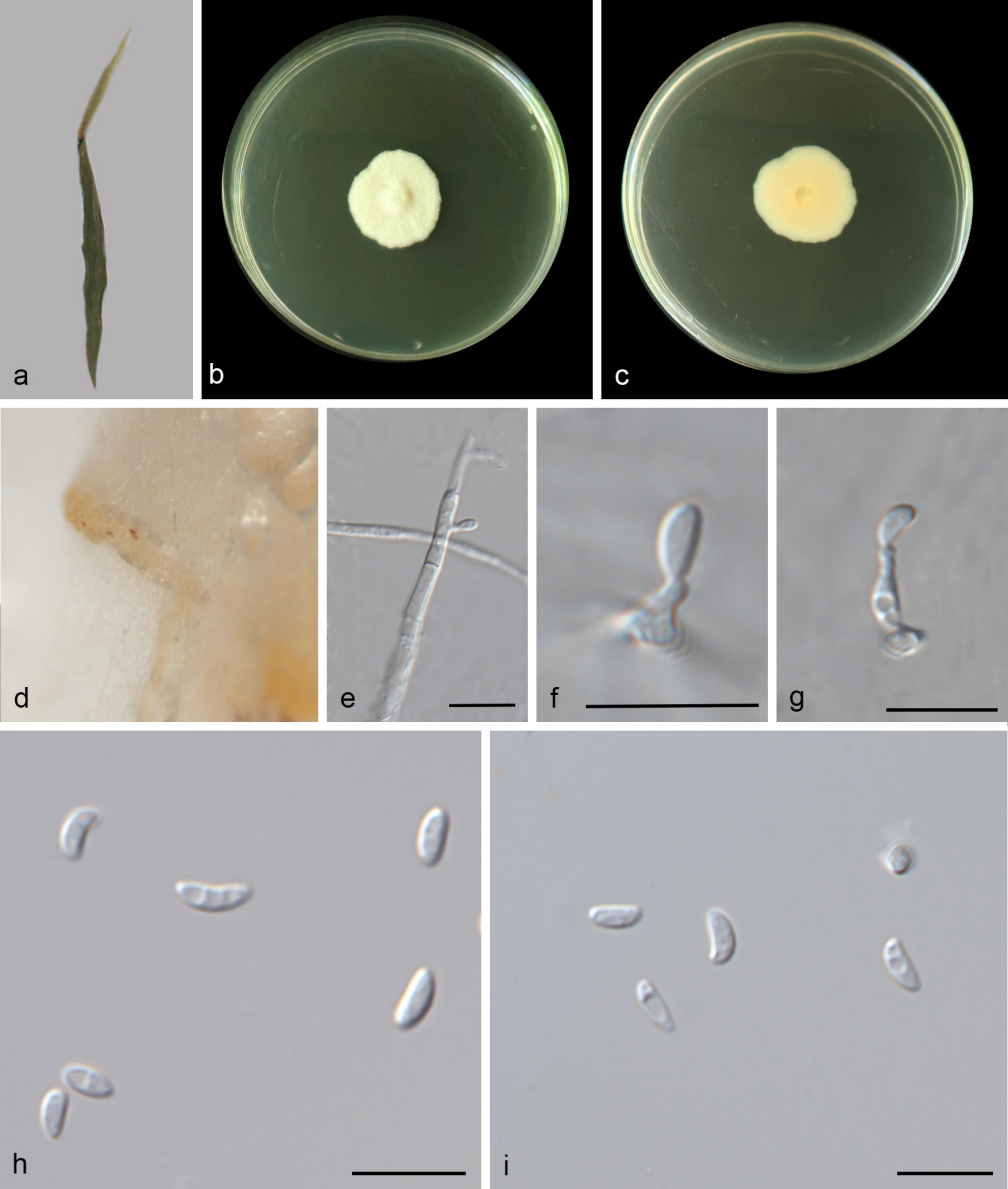
Morphology of *Microdochium setariae* (ex-type CGMCC 3.29420). **a** on leaf of *Setaria viridis***b, c** colonies (b-above, c-reverse) after 7 d on PDA**d** conidiomata formed on PDA**e–g** conidiogenous cells with conidia **h, i** conidia. Scale bars: 10 μm (**e–i**).

##### Type.

CHINA • Guangxi Zhuang Autonomous Region, Liuzhou City, Guting Mountain Forest Park, on leaves of *Setaria viridis*, 15 April 2025, Y.X. Shang (holotype HSAUP 18044-7), ex-type culture SAUCC 18044-7 = CGMCC 3.29420.

##### Description.

On leaves of *Setaria viridis*. Asexual morph: ***Mycelium*** mostly immersed, hyphae hyaline, septate, smooth, 1.6–3.3 μm wide. ***Conidiomata*** sporodochial, giving rise to orange conidial mass. ***Conidiophores*** reduced to conidiogenous cells. ***Conidiogenous cells*** terminal and intercalary, monoblastic, cylindrical to doliiform, 3.98–8.18 × 1.53–2.49 μm (av. = 5.38 ± 2.43 × 1.87 ± 0.54 μm, n = 11). ***Conidia*** lunate, fusiform or reniform, straight or curved, 4.17–6.66 × 1.86–2.76 μm (av. = 5.36 ± 0.69 × 2.16 ± 0.29 μm, n = 25). ***Chlamydospores*** not observed. Sexual morphs unknown, see Fig. 13.

##### Culture characteristics.

Colonies on PDA. 24–25 mm in diameter after 7 d at 25 °C in darkness, with a growth rate of 3.4–3.6 mm/day, undulate at edge, white in obverse, yellowish white in reverse.

##### Additional specimen examined.

CHINA • Guangdong Province, Guangzhou City, Conghua District, on diseased leaves of *Microstegium vimineum*, 01 May 2025, Y.X. Shang (HSAUP 17215-3), living culture SAUCC 17215-3. CHINA • Guangxi Zhuang Autonomous Region, Liuzhou City, Guting Mountain Forest Park, on diseased leaves of *Microstegium vimineum*, 15 April 2025, Y.X. Shang (HSAUP 18057-1), living culture SAUCC 18057-1.

##### Notes.

*Microdochium setariae* belongs to the large clade, where it shows a relationship with *M. viridis*, *M. chrysanthemoides*, and *M. novae-zelandiae* (Fig. 1). In phylogeny, *M. setariae* differs from *M. viridis* (SAUCC 18044-2) by 28 nucleotides (1/534 in ITS, 3/836 in LSU, and 24/841 in *rpb2*), differs from *M. chrysanthemoides* (CGMCC 3.17929) by 48 nucleotides (4/473 in ITS, 6/836 in LSU, and 38/841 in *rpb2*), and differs from *M. novae-zelandiae* (CPC 29376) by 49 nucleotides (4/534 in ITS, 5/693 in *tub2*, and 40/841 in *rpb2*). In morphology, *M. setariae* is distinguished from *M. viridis* by its mean conidia size (av. = 5.36 ± 0.69 × 2.16 ± 0.29 μm vs. av. = 8.31 ± 3.13 × 3.44 ± 0.75 μm), distinguished from *M. chrysanthemoides* by its conidiogenous cell width (1.53–2.49 μm vs. 3.0–4.5 μm) (Zhang et al. 2017), and distinguished from *M. novae-zelandiae* by its different conidiogenous cell type (monoblastic vs. polyblastic) (Marin-Felix et al. 2019). Therefore, based on morphology and phylogenetic evidence, we establish fungus as *Microdochium setariae* sp. nov.

#### Microdochium sinense

Taxon classificationFungiAmphisphaerialesAmphisphaeriaceae

S.B. Liu, X.Y. Liu, Z. Meng & X.G. Zhang, Journal of Fungi 8 (6), 577 (2022)

121BA199-0A2A-5DBB-BF0B-3A939C805D86

Fig. 14

##### Description.

**Figure 14.**
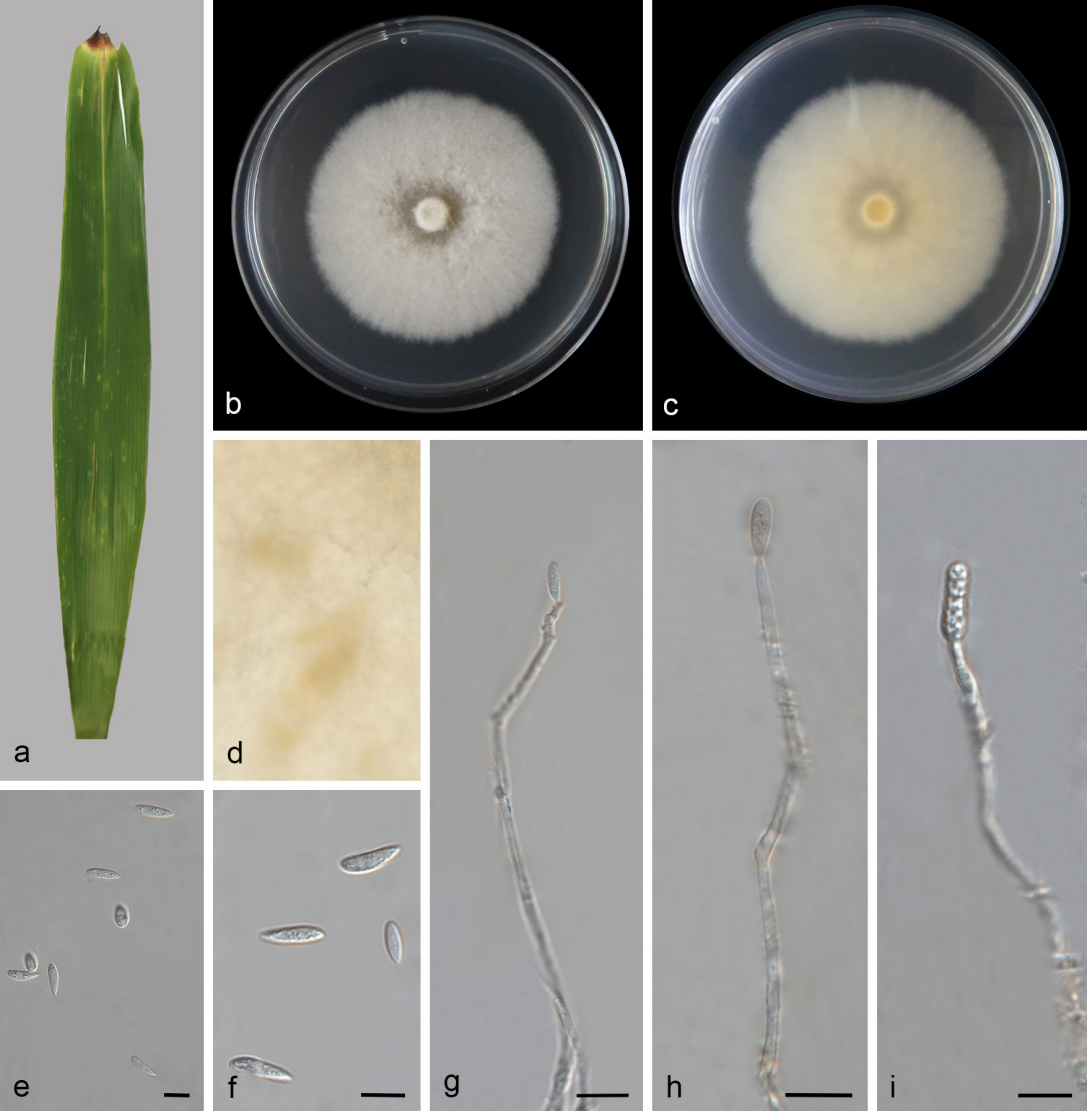
Morphology of *Microdochium sinense* (SAUCC 14457-1). **a** on leaf of *Thysanolaena latifolia***b, c** colonies (b-above, c-reverse) after 7 d on PDA**d** conidiomata formed on PDA**g–i** conidiophores with conidiogenous cells and conidia **e, f** conidia. Scale bars: 10 μm (**e–i**).

On leaves of *Thysanolaena latifolia*. Asexual morph: ***Mycelia*** are superficial and immersed, 1.3–2.1 µm wide, transparent, branched and diaphragmatic. ***Conidiophores*** are curved, produced from aerial hyphae, septate and often reduced to conidiogenous cells borne directly from hyphae, 79.45–104.52 × 2.39–2.82 µm. ***Conidiogenous cells*** are monoblastic, terminal, hyaline, smooth and cylindrical. ***Conidia*** are solitary, hyaline, spindle-shaped or cylindrical, 8.94–14.60 × 2.92–4.27 µm, guttulate when mature and sometimes borne directly from hyphae. ***Chlamydospores*** were not observed. Sexual morphs unknown, see Fig. 14.

##### Culture characteristics.

Colonies on PDA. 60.33–62.42 mm in diameter after 7 d at 25 °C in darkness, with a growth rate of 8.62–8.92 mm/day, entire at edge, white in obverse and pale yellow reverse.

##### Materials examined.

CHINA • Hainan Province, Ledong Li Autonomous County, Jianfengling National Forest Park, on leaves of *Thysanolaena latifolia*, 04 December 2024, Y.X. Shang (HSAUP 14457-1), living culture SAUCC 14457-1.

##### Distribution.

China, Hainan Province.

##### Ecology.

Associated with diseased leaves of *Thysanolaena latifolia* and *Miscanthus sinensis*.

##### Notes.

This isolate obtained from leaves of *Thysanolaena latifolia* formed a well-supported clade with two isolates of *Microdochium sinense* from *Miscanthus sinensis* leaves (Fig. 1; Liu et al. 2022). Hence, *Thysanolaena latifolia* become a new host for *M. sinense*.

#### Microdochium viridis

Taxon classificationFungiAmphisphaerialesAmphisphaeriaceae

Y.X. Shang, Q.Y. Liu, X.G. Zhang & Z. Li sp. nov.

B5F4F2A6-1766-5B70-8C62-E3EDF66E1D2A

Fungal Names: FN 573518

Fig. 15

##### Etymology.

The specific epithet “*viridis*” refers to the host plant *Setaria viridis*.

**Figure 15.**
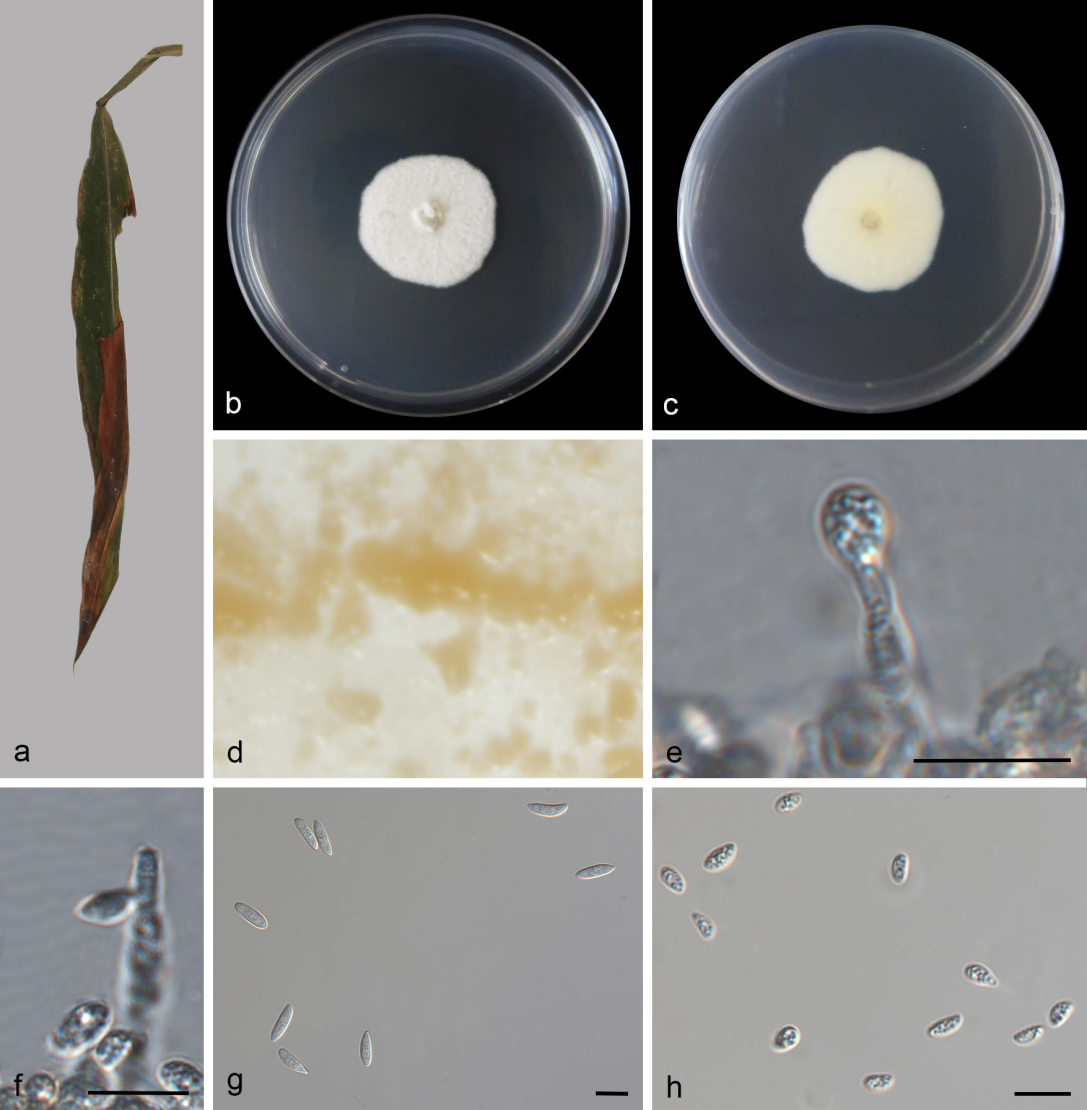
Morphology of *Microdochium viridis* (ex-type CGMCC 3.29434). **a** on leaf of *Setaria viridis***b, c** colonies (b-above, c-reverse) after 7 d on PDA**d** conidiomata formed on PDA**e, f** conidiogenous cells with conidia **g, h** conidia. Scale bars: 10 μm (**e–h**).

##### Type.

CHINA • Guangxi Zhuang Autonomous Region, Liuzhou City, Guting Mountain Forest Park, on leaves of *Setaria viridis*, 15 April 2025, Y.X. Shang (holotype HSAUP 18044-2), ex-type culture SAUCC 18044-2 = CGMCC 3.29434.

##### Description.

On leaves of *Setaria viridis*. Asexual morph: ***Mycelium*** mostly superficial, hyphae hyaline, septate. ***Conidiomata*** sporodochial, giving rise to pale orange conidial mass. ***Conidiophores*** reduced to conidiogenous cells. ***Conidiogenous cells*** terminal, monoblastic, cylindrical, 2.18–8.01 × 2.09–2.63 μm (av. = 5.55 ± 2.45 × 2.33 ± 0.24 μm, n = 14). ***Conidia*** fusiform or subglobose, 4.24–12.65 × 1.98–4.93 μm (av. = 8.31 ± 3.13 × 3.44 ± 0.75 μm, n = 18). ***Chlamydospores*** not observed. Sexual morphs unknown, see Fig. 15.

##### Culture characteristics.

Colonies on PDA. 31–33 mm in diameter after 7 d at 25 °C in darkness, with a growth rate of 4.4–4.7 mm/day, entire at edge, white in obverse and reverse.

##### Additional specimen examined.

CHINA • Guangxi Zhuang Autonomous Region, Liuzhou City, Guting Mountain Forest Park, on leaves of *Setaria viridis*, 15 April 2025, Y.X. Shang (HSAUP 18044-2A), living culture SAUCC 18044-2A.

##### Notes.

See *Microdochium setariae*.

#### Microdochium vulgaris

Taxon classificationFungiAmphisphaerialesAmphisphaeriaceae

Y.X. Shang, Q.Y. Liu, X.G. Zhang & Z. Li sp. nov.

B49FE1CA-38E0-5D96-9605-FD82EAE4DEE8

Fungal Names: FN 573519

Fig. 16

##### Etymology.

The specific epithet “*vulgaris*” refers to the host plant *Bambusa vulgaris*.

**Figure 16.**
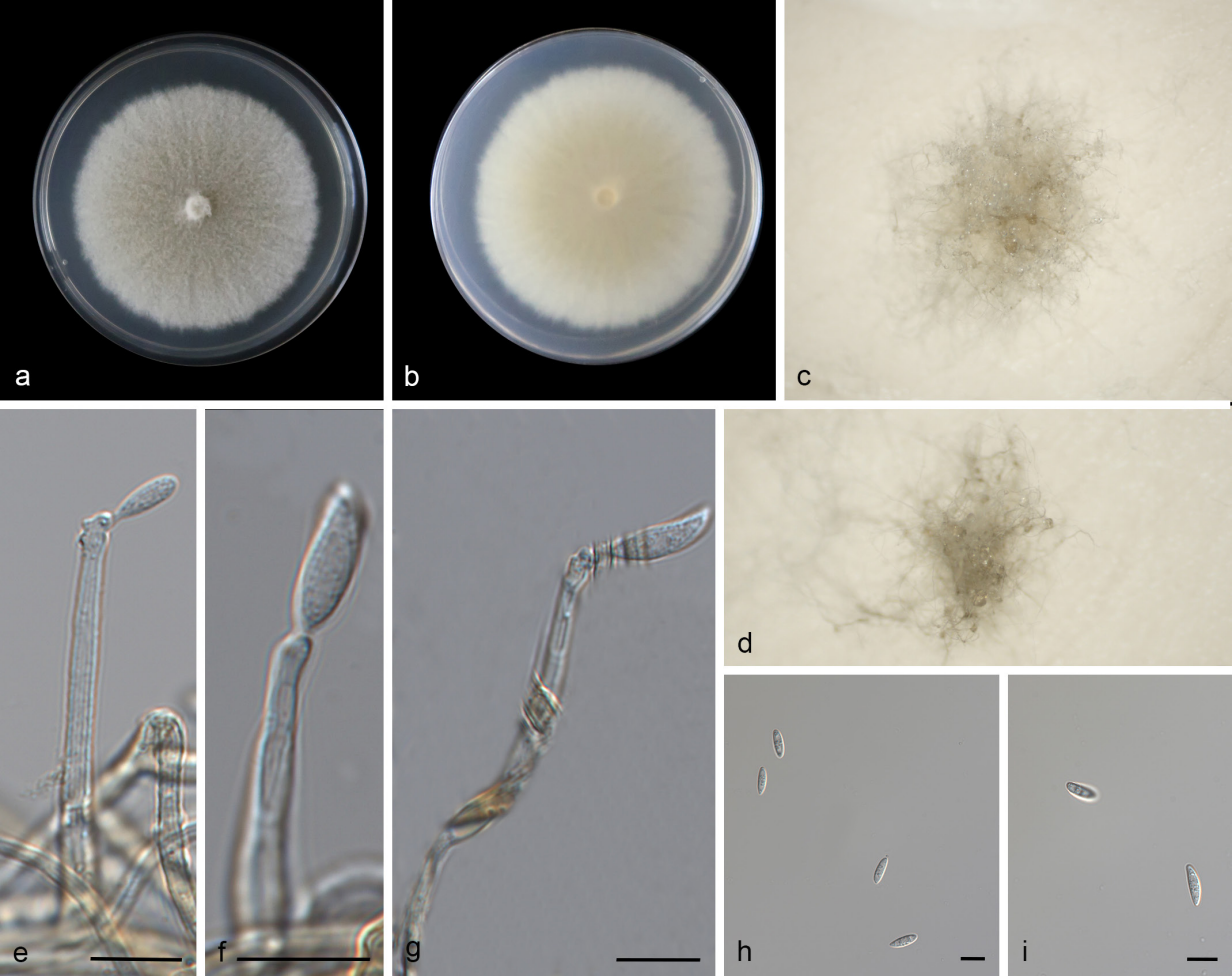
Morphology of *Microdochium vulgaris* (ex-type CGMCC 3.29422). **a, b** colonies (a-above, b-reverse) after 7 d on PDA**c, d** colony on PDA producing conidia masses **e–g** conidiophores with conidiogenous cells and conidia **h, i** conidia. Scale bars: 10 μm (**e–i**).

##### Type.

CHINA • Guangdong Province, Guangzhou City, Conghua District, on leaves of *Bambusa vulgaris*, 01 March 2025, Y.X. Shang (holotype HSAUP 17227-1), ex-type culture SAUCC 17227-1 = CGMCC 3.29422.

##### Description.

On leaves of *Bambusa vulgaris*. Asexual morph: ***Mycelium*** immersed and superficial, abundant, hyphae hyaline to pale brown, branched, septate, thin-walled, 2.02–3.28 µm wide. ***Conidiophores*** solitary, simple, rarely branched, straight or bent, cylindrical, often not clearly differentiated from the mycelium, hyaline to pale brown. ***Conidiogenous cells*** terminal, monoblastic, cylindrical, 25.17–54.47 × 2.65–3.27 μm (av. = 41.57 ± 14.69 × 2.97 ± 0.31 μm, n = 13). ***Conidia*** dry, reniform, fusiform, straight, smooth with thin walls, hyaline, 10.67–14.15 × 3.51–4.32 μm (av. = 11.84 ± 1.31 × 3.90 ± 0.33 μm, n = 16). ***Chlamydospores*** not observed. Sexual morphs unknown, see Fig. 16.

##### Culture characteristics.

Colonies on PDA. 70.20–73.58 mm in diameter after 7 d at 25 °C in darkness, with a growth rate of 10.03–10.51 mm/day, entire at edge, gray in obverse and fawn reverse.

##### Additional specimen examined.

CHINA • Guangdong Province, Guangzhou City, Conghua District, on leaves of *Bambusa vulgaris*, 01 March 2025, Y.X. Shang (HSAUP 17227-1A), living culture SAUCC 17227-1A.

##### Notes.

Nucleotide base pair comparisons across two gene regions (*rpb2* and *tub2*) between *M. vulgaris* and *M. bambusicola* (SAUCC 7623-2) species revealed differences of 29/841 bp and 12/702 bp. Nucleotide base pair comparisons across two gene regions (*rpb2* and *tub2*) between *M. vulgaris* and *M. bambusae* (SAUCC 1862-1) species revealed differences of 36/841 bp and 18/689 bp. In morphology, these species are distinguished by the length of conidiogenous cells in *M. vulgaris*, *M. bambusicola*, and *M. bambusae* (25.17–54.47 × 2.65–3.27 μm vs. 6.85–7.66 × 1.32–2.02 μm vs. 17.4–30.0 × 2.5–3.0 µm), and by their distinct conidial dimensions (10.67–14.15 × 3.51–4.32 μm vs. 6.28–11.12 × 2.85–4.11 μm vs. 13.0–17.0 × 2.5–3.5 µm) (Zhang et al. 2023a). Therefore, based on morphology and phylogenetic evidence, we establish this fungus as *Microdochium vulgaris* sp. nov.

## Discussion

Based on phylogenetic analyses, Hernández-Restrepo et al. (2016) proposed the new family *Microdochiaceae* to accommodate *Microdochium*, *Idriella*, and *Selenodriella*. Subsequently, *Peglionia*, *Xenoidriella*, and *Macroidriella* were proposed based on morphology and phylogenetic analyses (Hernández-Restrepo et al. 2022; Crous et al. 2023; Zhang et al. 2024). However, phylogenetic analyses and divergence time estimation based on a four-locus dataset (ITS, LSU, *rpb2*, and *tub2*) revealed that *Macroidriella* clusters within the genus *Microdochium*. Therefore, *Macroidriella* is synonymized under *Microdochium*.

Macrofungi and microfungi have been widely applied for biogeographical analyses (Zhao et al. 2022; Zhang et al. 2023b, 2023d; Mu et al. 2024). Our study suggests that the species distribution and speciation of *Microdochium* may originate in Asia, particularly in Southeast Asia. Divergence time of the *Microdochium* main clade emerged with a mean stem age of 56.0 Mya [95% HPD of 44.9–68.1 Mya] and a mean crown age of 49.5 Mya [95% HPD of 38.8–61.2 Mya], which belongs to the Paleogene period. This period was characterized by a globally warmer climate, with tropical to subtropical vegetation widely distributed, and land bridges connected Asia to Europe, while some speciation events linked Asia to Oceania. These connections facilitated the dispersal of fungi alongside their host plants into both Europe and Oceania. The main driver for the African to South America and Europe to Oceania dispersal route relies on LDD mechanisms, likely through the movement of spores via wind, ocean currents, weather patterns including hurricanes, monsoons, and other repeating large scale weather occurrences, as well as via animals and human migration (Golan and Pringle 2017). During the Neogene period, the Africa-Asia collision (via the Arabian Peninsula) facilitated dispersal from Asia to Africa. The collision of the Indian and Eurasian plates may have influenced species dispersal, vicariance, and extinction. Despite these advances, several taxonomic and evolutionary questions remain. Notably, tropical and subtropical regions—especially in Africa, Oceania, and South America—remain markedly undersampled and are confidently predicted to harbor significant undiscovered diversity within *Microdochium*.

Grounded in an integrative analysis of phylogenetic, morphological, and biogeographic data, this study proposes a stable and predictive five-genus system for *Microdochiaceae*, providing a robust taxonomic framework that also enables the reconstruction of historical biogeography. Thus, our work not only advances the classification of this family but also offers testable hypotheses regarding the origin and dispersal of *Microdochium*.

## Supplementary Material

XML Treatment for Microdochium bambusicola

XML Treatment for Microdochium

XML Treatment for Microdochium danzhouense

XML Treatment for Microdochium guangdongense

XML Treatment for Microdochium guizhouensis

XML Treatment for Microdochium ledongense

XML Treatment for Microdochium microstegii

XML Treatment for Microdochium miscanthi

XML Treatment for Microdochium nannuoshanense

XML Treatment for Microdochium nigrum

XML Treatment for Microdochium setariae

XML Treatment for Microdochium sinense

XML Treatment for Microdochium viridis

XML Treatment for Microdochium vulgaris

## Additional information

### Artificial Intelligence (AI) use

The authors accept full responsibility for the content of the manuscript, including the disclosure of any use of AI.

No AI tools were used in the preparation of this manuscript.

### Adherence to national and international regulations

All the fungal strains used in this study have been legally obtained, respecting the Convention on Biological Diversity (Rio Convention).

### Author ORCIDs

Q. Liu https://orcid.org/0009-0009-9545-7962

Y. Shang https://orcid.org/0009-0000-7211-5139

Z. Zhang https://orcid.org/0000-0002-4824-9716

J. Xia https://orcid.org/0000-0002-7436-7249

X. Zhang https://orcid.org/0000-0001-9733-8494

Z. Li https://orcid.org/0000-0003-0583-0399

### Data availability

All of the data that support the findings of this study are available in the main text or Supplementary Information.

## Supplementary materials

Supplementary material 1 Species information, collection localities, and GenBank accession numbers for *Microdochium* sequences used in divergence time estimation and historical biogeographic analysis Data type pdf https://binary.pensoft.net/file/1677532This dataset is made available under the Open Database License (http://opendatacommons.org/licenses/odbl/1.0/). The Open Database License (ODbL) is a license agreement intended to allow users to freely share, modify, and use this Dataset while maintaining this same freedom for others, provided that the original source and author(s) are credited. Qiyun Liu, Yuxin Shang, Jinyang Yuan, Zhaoxue Zhang, Congcong Ai, Jiwen Xia, Xiuguo Zhang, Zhuang Li

Supplementary material 2 Single-locus phylograms of the family *Microdochiaceae* based on alignments of the ITS, LSU, *rpb2*, and *tub2* regions, respectively Data type pdf https://binary.pensoft.net/file/1677533This dataset is made available under the Open Database License (http://opendatacommons.org/licenses/odbl/1.0/). The Open Database License (ODbL) is a license agreement intended to allow users to freely share, modify, and use this Dataset while maintaining this same freedom for others, provided that the original source and author(s) are credited. Qiyun Liu, Yuxin Shang, Jinyang Yuan, Zhaoxue Zhang, Congcong Ai, Jiwen Xia, Xiuguo Zhang, Zhuang Li

Supplementary material 3 An alignment used in this study Data type fas https://binary.pensoft.net/file/1677534This dataset is made available under the Open Database License (http://opendatacommons.org/licenses/odbl/1.0/). The Open Database License (ODbL) is a license agreement intended to allow users to freely share, modify, and use this Dataset while maintaining this same freedom for others, provided that the original source and author(s) are credited. Qiyun Liu, Yuxin Shang, Jinyang Yuan, Zhaoxue Zhang, Congcong Ai, Jiwen Xia, Xiuguo Zhang, Zhuang Li
