# Supplementary material for: Multilocus phylogeny, morphology and taxonomy of Microdochium (Microdochiaceae): insights into evolutionary divergence times and historical biogeography
Source: IMA Fungus. 2026 Jun 12;17:e191909. doi: 10.3897/imafungus.17.191909 (PMC13282624; doi:10.3897/imafungus.17.191909)
Supplement: Supplementary material 1 — Species information, collection localities, and GenBank accession numbers for Microdochium sequences used in divergence time estimation and historical biogeographic analysis [file imafungus-17-e191909-s001.pdf]

Supplementary material 1. Species information, collection localities, and GenBank accession numbers of sequences used for divergence time estimation and historical biogeographic analysis

Species	Strains	Country	ITS	LSU	*rpb2*	*tub2*	References
Arecophila bambusae	HKUCC 4794	Unknown country	N/A	AF452038	N/A	N/A	(Jeewon et al. 2003)
Biscogniauxia nummularia	MUCL 51395	France	KY610382	KY610427	KY624236	KX271241	(Wendt et al. 2017)
Cainia graminis	CBS 136.62	France	MH858123	MH869701	N/A	N/A	(Vu et al. 2019)
Calosphaeria africana	STE-U 6181	South Africa	EU367445	EU367455	N/A	EU367465	(Damm et al. 2008)
Capnodium paracoartatum	MFLU 19-2888	Thailand	MT177926	MT177953	N/A	N/A	(Li et al. 2020)
Colletotrichum boninense	CBS 123755	Japan	MH863323	MH874855	N/A	JQ005588	(Moriwaki et al. 2003; Damm et al. 2012)
Coniochaeta arenariae	MFLUCC 18-0405	UK	MN047126	MN017896	N/A	N/A	(Dayarathne 2020)
Diatrype disciformis	AFTOL-ID 927	USA	N/A	N/A	DQ470915	N/A	(Spatafora et al. 2017)
Eutypa lata	CBS 208.87	Switzerland	MH862066	MH873755	N/A	DQ006969	(Rolshausen et al. 2006)
Graphostroma platystoma	AFTOL-ID 1249	USA	N/A	DQ836906	DQ836893	N/A	(Zhang et al. 2017)
Idriella chlamydospora	CGMCC 3.20778	China	OL897016	OL897058	N/A	ON569069	(Zhang et al. 2023c)
Idriella multiformispora	CGMCC 3.20779	China	OL897018	OL897060	N/A	ON569071	

Iodosphaeria tongrenensis	MFLU 15-0393	China	KR095282	KR095283	N/A	N/A	(Li et al. 2015)
Kretzschmaria deusta	CBS 163.93	Germany	KC477237	KY610458	KY624227	KX271251	(Wendt et al. 2017)
Microdochium danzhouensis	SAUCC 6792-1	China	PP716851	PP716512	PP729053	PP729058	This study
Magnaporthiopsis agrostidis	BRIP 59300	Australia	KT364753	KT364754	N/A	N/A	(Crous et al. 2015)
Metacapnodium neesii	JCM 39119	Japan	LC576698	LC576694	N/A	N/A	(Sugiyama et al. 2020)
Microdochium albescens	CBS 291.79	Ivory Coast	KP858996	KP858932	KP859105	KP859059	(Hernández-Restrepo et al. 2016)
Microdochium albescens	CBS 243.83	Unknown country	KP858994	KP858930	KP859103	KP859057	
Microdochium australe	SAUCC 6322-5-1	China	PP695312	PP702043	PP716780	PP716787	(Zhang et al. 2024)
Microdochium australiana	SAUCC 6340-2-6	China	PQ807110	PV609100	PV975978	PV686755	(Shang et al. 2025)
Microdochium baishamenense	SAUCC 8129-1	China	PQ807114	PV609104	PV975982	PV686759	
Microdochium bambooensis	SAUCC 7623-2 = CGMCC 3.29432	China	PX569617	PX578006	PX705309	PX705279	This study
Microdochium bambusae	SAUCC 1862-1	China	OR702567	OR702576	OR715785	PP445175	(Zhang et al. 2023a)

Microdochium bambusarum	SAUCC 7611-3	China	PQ807112	PV609102	PV975980	PV686757	(Shang et al. 2025)
Microdochium bambusina	SAUCC 7531-3	China	PQ807108	PV609098	PV975976	PV686753	(Shang et al. 2025)
Microdochium bolleyi	CBS 540.92	Syria	KP859010	KP858946	KP859119	KP859073	(Hernández-Restrepo et al. 2016)
Microdochium buffelskloofinum	SA1SD	South Africa	PP791437.1	PP791465.1	PP780615.1	PP780639.1	(Crous et al. 2024)
Microdochium chrysanthemoides	CGMCC 3.17929	China	KU746690	KU746736	N/A	N/A	(Zhang et al. 2017)
Microdochium chrysopogonis	GDMCC 3.683	China	MT988022	MT988024	MW002444	MW002441	(Lu et al. 2023)
Microdochium chuxiongense	YFCC 8794	China	OK586161	OK586160	OK584019	OK556901	(Tang et al. 2022)
Microdochium citrinidiscum	CBS 109067	Peru	KP859003	KP858939	KP859112	KP859066	(Hernández-Restrepo et al. 2016)
Microdochium colombiense	CBS 624.94	Colombia	KP858999	KP858935	KP859108	KP859062	
Microdochium dawsoniorum	BRIP 65649	Australia	MK966337	N/A	N/A	N/A	(Crous et al. 2020)
Microdochium fisheri	CBS 242.90	UK	KP859015	KP858951	KP859124	KP859079	(Hernández-Restrepo et al. 2016)
Microdochium gongcheniae	GDMCC3.1048 = YNE01164	China	PP111926	PP111933	N/A	PP112586	(Yan and Zhang 2024)

Microdochium graminearum	CGMCC 3.23525	China	OP103966	OP104016	OP236027	N/A	(Gao et al. 2022)
Microdochium graminis	GDMCC3.1049	China	PP111928	PP111935	PP112593	PP112588	(Yan and Zhang 2024)
Microdochium guangdongensis	SAUCC 17239-1 = CGMCC 3.29429	China	PX569636	PX578025	PX705328	PX705298	This study
Microdochium guizhouensis	GUCC 25–0012	China	N/A	N/A	PV505431	PV505433	(Hongsanan et al. 2025)
Microdochium hainanense	SAUCC 210781	China	OM956295	OM959323	OM981153	OM981146	(Liu et al. 2022)
Microdochium hongkuii	GDMCC3.1079 = YNE00483	China	PP111923	PP111930	PP112590	PP112583	(Yan and Zhang 2024)
Microdochium indocalami	SAUCC 1016	China	MT199884	MT199878	MT510550	MT435653	(Huang et al. 2020)
Microdochium jianfenglingense	SAUCC 1862-2	China	PP702394	PP711783	PP716793	PP716799	(Li et al. 2024)
Microdochium ledongensis	SAUCC 14499-3	China	PX569628	PX578017	PX705319	PX705289	This study
Microdochium lycopodinum	CBS 122885	Austria	KP859016	KP858952	KP859125	KP859080	(Hernández-Restrepo et al. 2016)
Microdochium maculosum	COAD 3358	Brazil	Ok966954	Ok966953	N/A	N/A	(Crous et al. 2021b)
Microdochium microstegium	SAUCC 14288-1	China	PX569626	PX578015	PX705317	PX705287	This study

Microdochium miscanthi	SAUCC 211092	China	OM956214	OM957532	OM981148	OM981141	(Liu et al. 2022)
Microdochium musae	CBS 143500	Malaysia	MH107895	MH107942	MH108003	N/A	(Crous et al. 2018)
Microdochium nannuoshanense	SAUCC 2450-1	China	OR702569	OR702578	OR715787	PP445177	(Zhang et al. 2023a)
Microdochium neoqueenslandicum	CBS 108926	New Zealand	KP859002	KP858938	KP859111	KP859065	(Hernández-Restrepo et al. 2016)
Microdochium nigra	SAUCC 17349-2 = CGMCC 3.29428	China	PX569638	PX578027	PX705330	PX705300	This study
Microdochium nivale	CBS 116205	UK	KP859008	KP858944	KP859117	KP859071	(Hernández-Restrepo et al. 2016)
Microdochium novae-zelandiae	CPC 29376	New Zealand	LT990655	N/A	LT990641	LT990608	(Marin-Felix et al. 2019)
Microdochium oryzicola	MFLUCC 24-0509	Thailand	PV241406	PV241407	PV275683	N/A	(Absalan et al. 2025)
Microdochium paspali	CBS 138620	China	KJ569513	N/A	N/A	KJ569518	(Zhang et al. 2015)
Microdochium phragmitis	CBS 285.71	Poland	KP859013	KP858949	KP859122	KP859077	(Hernández-Restrepo et al. 2016)
Microdochium phyllosaprophyticum	SAUCC 3583-1	China	OR702571	OR702580	OR715789	PP445179	(Zhang et al. 2023a)
Microdochium poae	CGMCC 3.19170	China	MH740898	N/A	MH740906	MH740914	(Liang et al. 2019)

Microdochium ratticaudae	BRIP 68298	Australia	MW481661	MW481666	MW626890	N/A	(Crous et al. 2021a)
Microdochium rhopalostylidis	CBS 145125	New Zealand	MK442592	MK442532	MK442667	N/A	(Crous et al. 2019)
Microdochium salmonicolor	NC14-294	Korea	MK836110	MK836108	N/A	N/A	(Das et al. 2020)
Microdochium seminicola	CBS 139951	Switzerland	KP859038	KP858974	KP859147	KP859101	(Hernández-Restrepo et al. 2016)
Microdochium setariae	SAUCC 18044-7 = CGMCC 3.29420	China	PX569644	PX578033	PX705335	PX705305	This study
Microdochium shilinense	CGMCC 3.23531	China	OP103972	OP104022	N/A	OP242834	(Gao et al. 2022)
Microdochium sichuanense	KUNCC23-13008	China	OQ616510	OQ616434	OQ623473	N/A	(Dissanayake et al. 2023)
Microdochium sinense	SAUCC 211097	China	OM956289	OM959225	OM981151	OM981144	(Liu et al. 2022)
Microdochium streetiae	BRIP 74742a	Australia	OR947072	OR947079	N/A	N/A	(Tan and Shivas 2023b)
Microdochium tainanense	CBS 269.76	China	KP859009	KP858945	KP859118	KP859072	(Hernández-Restrepo et al. 2016)
Microdochium trichocladiopsis	CBS 623.77	Unknown country	KP858998	KP858934	KP859107	KP859061	
Microdochium triticicola	RR 241	UK	AJ748691	N/A	N/A	N/A	(Kwasna and Bateman 2007)

Microdochium viridis	SAUCC 18044-2	China	PX569642	PX578030	PX705333	PX705303	This study
Microdochium vulgaris	SAUCC 17227-1	China	PX569634	PX578023	PX705326	PX705296	This study
Microdochium yunnanense	SAUCC 1011	China	MT199881	MT199875	MT510547	MT435650	(Huang et al. 2020)
Nakazawaea tricholoma	CGMCC 2.7006	China	OQ179817	N/A	N/A	N/A	(Liu et al. 2024)
Neurospora crassa	OR74A	India	HQ271348	AF286411	AF107789	N/A	(Untereiner et al. 2001)
Ophiostoma ainoae	CBS 205.83	Norway	MH861571	MH873301	N/A	N/A	(Yamaoka et al. 1997)
Peglionia falcat	GUCC 23-0042	China	PP295269	PP314032	PP396044	N/A	(Fu et al. 2024)
Phaeoacremonium adelophialidum	P30	Algeria	MW689543	MW689544	N/A	N/A	(Crous et al. 2021a)
Phyllachora isachnicola	MHYAU 179	China	MH018561	MH018563	N/A	N/A	(Li et al. 2018)
Rosellinia necatrix	Rn-6	China	OR511446	OR511447	N/A	OR544809	From NCBI
Selenodriella brasiliana	CBS 140227= MUCL 41176	Brazil	ON400769	ON400821	ON399356	N/A	(Hernández-Restrepo et al. 2022)
Selenodriella cubensis	CBS 683.96= INIFAT C96/30	Cuba	KP859053.1	KP858990.1	N/A	N/A	(Hernández-Restrepo et al. 2016)
Selenodriella fertilis	CBS 772.83	Netherlands	KP859055	MH873401	N/A	N/A	
Seynesia erumpens	SMH 1291	Unknown country	N/A	AF279410	AY641073	N/A	(Bhattacharya et al. 2000)
Xenoidriella cinnamomi	CPC 43130 = CBS 149458	South africa	OQ628471.1	OQ629053.1	OQ627937.1	N/A	(Crous et al. 2023)

Xylaria longipes	CBS 148.73	Germany	MH860649	MH872351	KU684280	KU684204	(Vu et al. 2019)

Note. NA means not available.

References

Bhattacharya D, Lutzoni F, Reeb V et al. (2000) Widespread Occurrence of Spliceosomal Introns in the rDNA Genes of *Ascomycetes*. Molecular Biology and Evolution 17(12): 1971–1984. https://doi.org/10.1093/oxfordjournals.molbev.a026298

Crous PW, Wingfield MJ, Roux JJL et al. (2015) Fungal Planet description sheets: 371–399. Persoonia 35: 264–327. https://doi.org/10.3767/003158515x690269

Damm U, Cannon PF, Woudenberg JH et al. (2012) The *Colletotrichum boninense* species complex. Studies in Mycology 73: 1–36. https://doi.org/10.3114/sim0002

Damm U, Crous PW, Fourie PH (2008) A fissitunicate ascus mechanism in the *Calosphaeriaceae*, and novel species of *Jattaea* and *Calosphaeria* on *Prunus* wood. Persoonia 20: 39–52. https://doi.org/10.3767/003158508X313940

Dayarathne MC (2020) Morpho-molecular characterization of microfungi associated with marine based habitats. Mycosphere 11: 1–188. https://doi.org/10.5943/mycosphere/11/1/1

Jeewon R, Liew EC, Hyde KD (2003) Molecular systematics of the *Amphisphaeriaceae* based on cladistic analyses of partial LSU rDNA gene sequences. Mycological Research 107: 1392–1402. https://doi.org/10.1017/s095375620300875x

Li Q-R, Kang J-C, Hyde KD (2015) A multiple gene genealogy reveals the phylogenetic placement of *Iodosphaeria tongrenensis* sp. nov. in *Iodosphaeriaceae* (*Xylariales*). Phytotaxa 234(2): 121–132. http://dx.doi.org/10.11646/phytotaxa.234.2.2

Li W-J, McKenzie EHC, Liu J-K et al. (2020) Taxonomy and phylogeny of hyaline-spored coelomycetes. Fungal Diversity 100: 279–801. https://doi.org/10.1007/s13225-020-00440-y

Li X-L, Yang Z-X, Wang X-X et al. (2018) Two new species of graminicolous *Phyllachora* (*Phyllachoraceae*, *Ascomycota*). Mycosystema 37(9): 1127–1132. https://doi.org/10.13346/j.mycosystema.180054

Liu M, Jiang YL, Zhang YX et al. (2024) *Nakazawaea tricholomae* f.a., sp. nov., a Novel Ascomycetous Yeast Species Isolated from Two Mushroom Species in China. Current Microbiology 81: 78. https://doi.org/10.1007/s00284-023-03600-w

Moriwaki J, Sato T, Tsukiboshi T (2003) Morphological and molecular characterization of *Colletotrichum boninense* sp. nov. from Japan. Mycoscience 44: 47–53. https://doi.org/10.1007/s10267-002-0079-7

Rolshausen PE, Mahoney NE, Molyneux RJ et al. (2006) A Reassessment of the Species Concept in *Eutypa lata*, the Causal Agent of Eutypa Dieback of Grapevine. Mycology 96(4): 369–377. https://doi.org/10.1094/PHYTO-96-0369

Spatafora JW, Sung G-H, Johnson D et al. (2017) A five-gene phylogeny of *Pezizomycotina*. Mycologia 98: 1018–1028. https://doi.org/10.1080/15572536.2006.11832630

Sugiyama J, Nam K-O, Hosoya T (2020) *Metacapnodium neesii*: a new combination for a metacapnodiaceous sooty mould and its phylogenetic position inferred from DNA sequences. Journal of Fungal Research 18(04): 246–257. https://doi.org/10.13341/j.jfr.2020.8002

Untereiner WA, Débois V, Naveau FA (2001) Molecular systematics of the ascomycete genus *Farrowia* (*Chaetomiaceae*). Canadian Journal of Botany 79: 321–333. https://doi.org/10.1139/cjb-79-3-321

Wendt L, Sir EB, Kuhnert E et al. (2017) Resurrection and emendation of the *Hypoxylaceae*, recognised from a multigene phylogeny of the *Xylariales*. Mycological Progress 17: 115–154. https://doi.org/10.1007/s11557-017-1311-3

Yamaoka Y, Wingfield MJ, Takahashi I et al. (1997) Ophiostomatoid fungi associated with the spruce bark beetle *Ips typographus* f. *aponicus* in Japan. Mycological Research 101: 1215–1227. https://doi.org/10.1017/s0953756297003924

Zhang N, Castlebury LA, Miller AN et al. (2017) An overview of the systematics of the *Sordariomycetes* based on a four-gene phylogeny. Mycologia 98: 1076–1087. https://doi.org/10.1080/15572536.2006.11832635
