# Supplementary material for: Multilocus phylogeny, morphology and taxonomy of Microdochium (Microdochiaceae): insights into evolutionary divergence times and historical biogeography
Source: IMA Fungus. 2026 Jun 12;17:e191909. doi: 10.3897/imafungus.17.191909 (PMC13282624; doi:10.3897/imafungus.17.191909)
Supplement: Supplementary material 2 — Single-locus phylograms of the family Microdochiaceae based on alignments of the ITS, LSU, rpb2, and tub2 regions, respectively [file imafungus-17-e191909-s002.pdf]

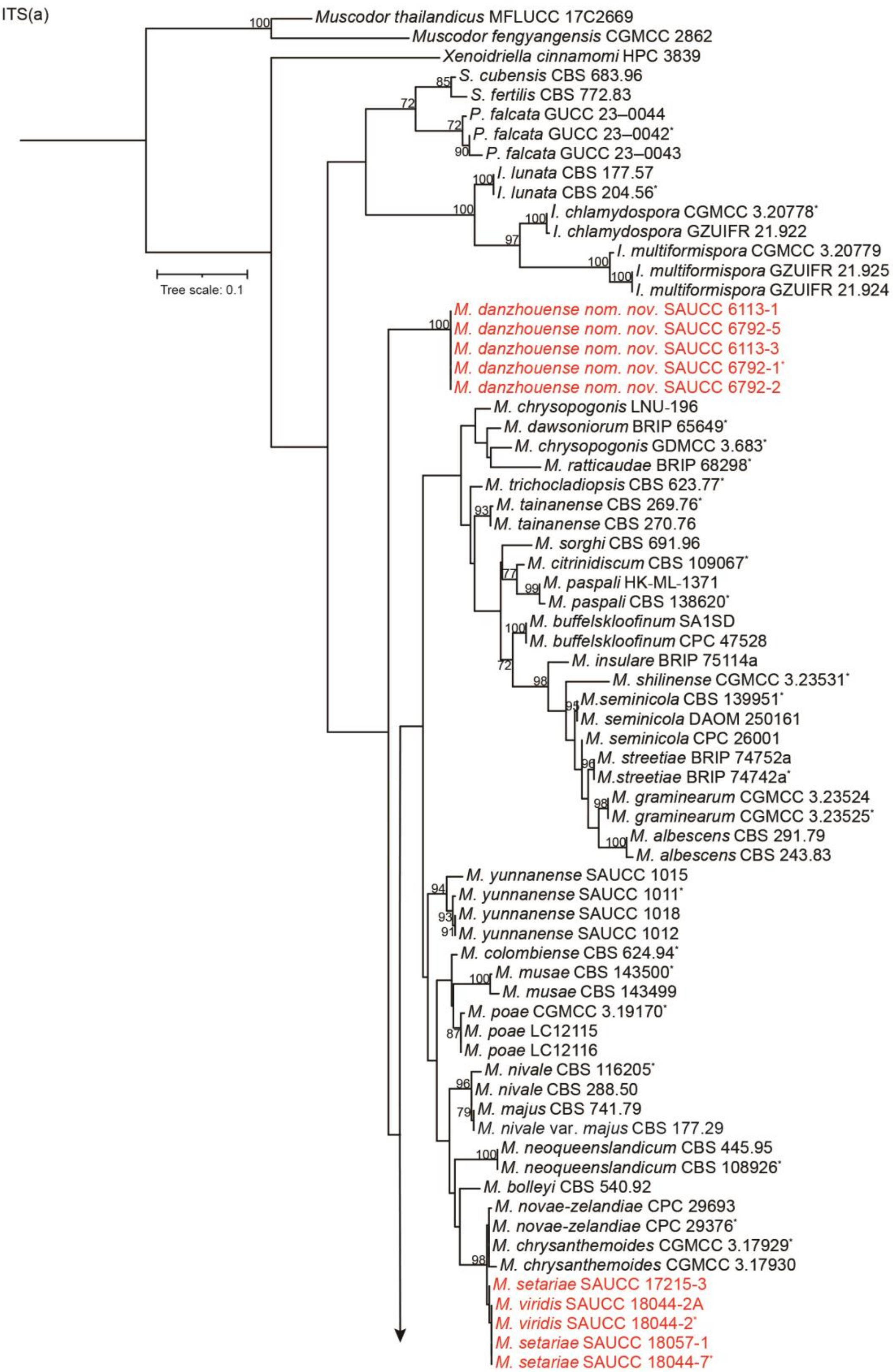
ITS(a)
Muscodor thailandicus MFLUCC 17C2669
Muscodor fengyangensis CGMCC 2862
Xenoidriella cinnamomi HPC 3839
S. cubensis CBS 683.96
S. fertilis CBS 772.83
P. falcata GUCC 23–0044
P. falcata GUCC 23–0042*
P. falcata GUCC 23–0043
I. lunata CBS 177.57
I. lunata CBS 204.56*
I. chlamydospora CGMCC 3.20778*
I. chlamydospora GZUIFR 21.922
I. multiformispora CGMCC 3.20779
I. multiformispora GZUIFR 21.925
I. multiformispora GZUIFR 21.924
Tree scale: 0.1
M. danzhouense nom. nov. SAUCC 6113-1
M. danzhouense nom. nov. SAUCC 6792-5
M. danzhouense nom. nov. SAUCC 6113-3
M. danzhouense nom. nov. SAUCC 6792-1*
M. danzhouense nom. nov. SAUCC 6792-2
M. chrysopogonis LNU-196
M. dawsoniorum BRIP 65649*
M. chrysopogonis GDMCC 3.683*
M. ratticaudae BRIP 68298*
M. trichocladiopsis CBS 623.77*
M. tainanense CBS 269.76*
M. tainanense CBS 270.76
M. sorghi CBS 691.96
M. citrinidiscum CBS 109067*
M. paspali HK-ML-1371
M. paspali CBS 138620*
M. buffelskloofinum SA1SD
M. buffelskloofinum CPC 47528
M. insulare BRIP 75114a
M. shilinense CGMCC 3.23531*
M.seminicola CBS 139951*
M. seminicola DAOM 250161
M. seminicola CPC 26001
M. streetiae BRIP 74752a
M.streetiae BRIP 74742a*
M. graminearum CGMCC 3.23524
M. graminearum CGMCC 3.23525*
M. albescens CBS 291.79
M. albescens CBS 243.83
M. yunnanense SAUCC 1015
M. yunnanense SAUCC 1011*
M. yunnanense SAUCC 1018
M. yunnanense SAUCC 1012
M. colombiense CBS 624.94*
M. musae CBS 143500*
M. musae CBS 143499
M. poae CGMCC 3.19170*
M. poae LC12115
M. poae LC12116
M. nivale CBS 116205*
M. nivale CBS 288.50
M. majus CBS 741.79
M. nivale var. majus CBS 177.29
M. neoqueenslandicum CBS 445.95
M. neoqueenslandicum CBS 108926*
M. bolleyi CBS 540.92
M. novae-zelandiae CPC 29693
M. novae-zelandiae CPC 29376*
M. chrysanthemoides CGMCC 3.17929*
M. chrysanthemoides CGMCC 3.17930
M. setariae SAUCC 17215-3
M. viridis SAUCC 18044-2A
M. viridis SAUCC 18044-2*
M. setariae SAUCC 18057-1
M. setariae SAUCC 18044-7*

ITS(b)

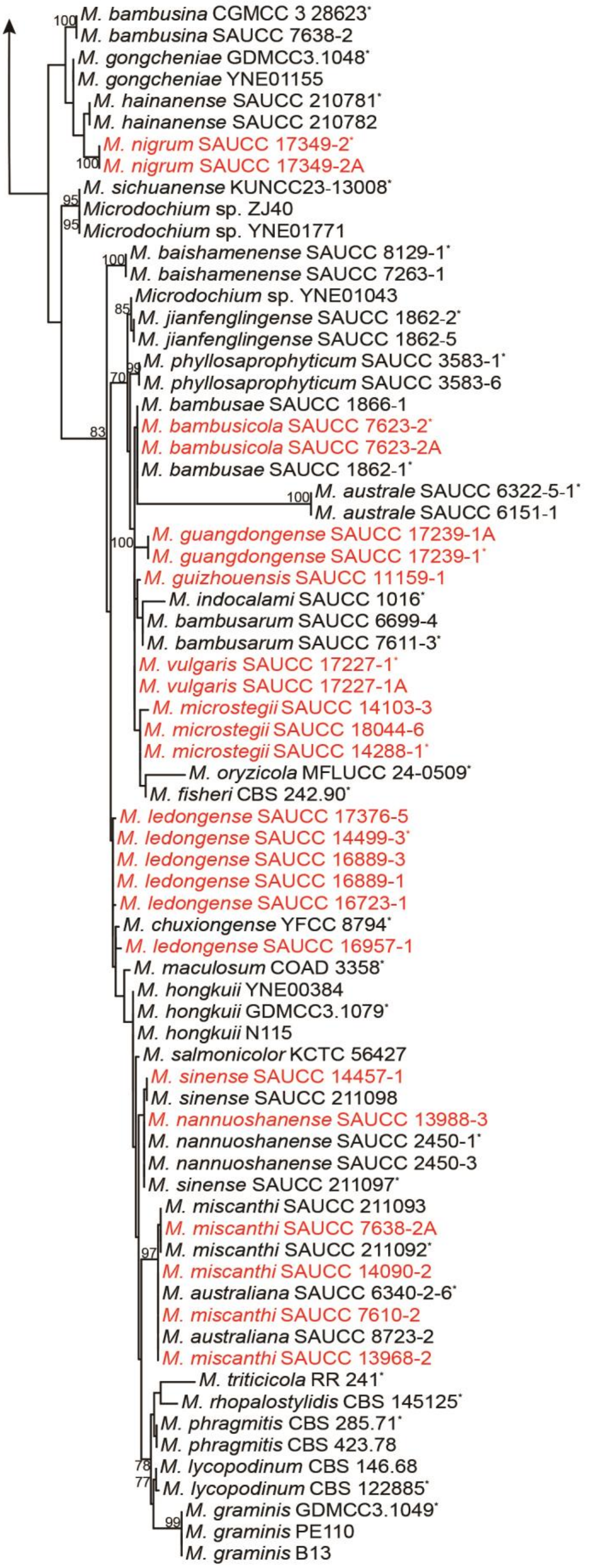
M. bambusina CGMCC 3 28623*
M. bambusina SAUCC 7638-2
M. gongcheniae GDMCC3.1048*
M. gongcheniae YNE01155
M. hainanense SAUCC 210781*
M. hainanense SAUCC 210782
M. nigrum SAUCC 17349-2*
M. nigrum SAUCC 17349-2A
M. sichuanense KUNCC23-13008*
Microdochium sp. ZJ40
Microdochium sp. YNE01771
M. baishamenense SAUCC 8129-1*
M. baishamenense SAUCC 7263-1
Microdochium sp. YNE01043
M. jianfenglingense SAUCC 1862-2*
M. jianfenglingense SAUCC 1862-5
M. phyllosaprophyticum SAUCC 3583-1*
M. phyllosaprophyticum SAUCC 3583-6
M. bambusae SAUCC 1866-1
M. bambusicola SAUCC 7623-2*
M. bambusicola SAUCC 7623-2A
M. bambusae SAUCC 1862-1*
M. australe SAUCC 6322-5-1*
M. australe SAUCC 6151-1
M. guangdongense SAUCC 17239-1A
M. guangdongense SAUCC 17239-1*
M. guizhouensis SAUCC 11159-1
M. indocalami SAUCC 1016*
M. bambusarum SAUCC 6699-4
M. bambusarum SAUCC 7611-3*
M. vulgaris SAUCC 17227-1*
M. vulgaris SAUCC 17227-1A
M. microstegii SAUCC 14103-3
M. microstegii SAUCC 18044-6
M. microstegii SAUCC 14288-1*
M. oryzicola MFLUCC 24-0509*
M. fisheri CBS 242.90*
M. ledongense SAUCC 17376-5
M. ledongense SAUCC 14499-3*
M. ledongense SAUCC 16889-3
M. ledongense SAUCC 16889-1
M. ledongense SAUCC 16723-1
M. chuxiongense YFCC 8794*
M. ledongense SAUCC 16957-1
M. maculosum COAD 3358*
M. hongkuii YNE00384
M. hongkuii GDMCC3.1079*
M. hongkuii N115
M. salmonicolor KCTC 56427
M. sinense SAUCC 14457-1
M. sinense SAUCC 211098
M. nannuoshanense SAUCC 13988-3
M. nannuoshanense SAUCC 2450-1*
M. nannuoshanense SAUCC 2450-3
M. sinense SAUCC 211097*
M. miscanthi SAUCC 211093
M. miscanthi SAUCC 7638-2A
M. miscanthi SAUCC 211092*
M. miscanthi SAUCC 14090-2
M. australiana SAUCC 6340-2-6*
M. miscanthi SAUCC 7610-2
M. australiana SAUCC 8723-2
M. miscanthi SAUCC 13968-2
M. triticicola RR 241*
M. rhopalostylidis CBS 145125*
M. phragmitis CBS 285.71*
M. phragmitis CBS 423.78
M. lycopodinum CBS 146.68
M. lycopodinum CBS 122885*
M. graminis GDMCC3.1049*
M. graminis PE110
M. graminis B13
100
100
95
95
100
85
99
70
83
100
100
97
78
77
99

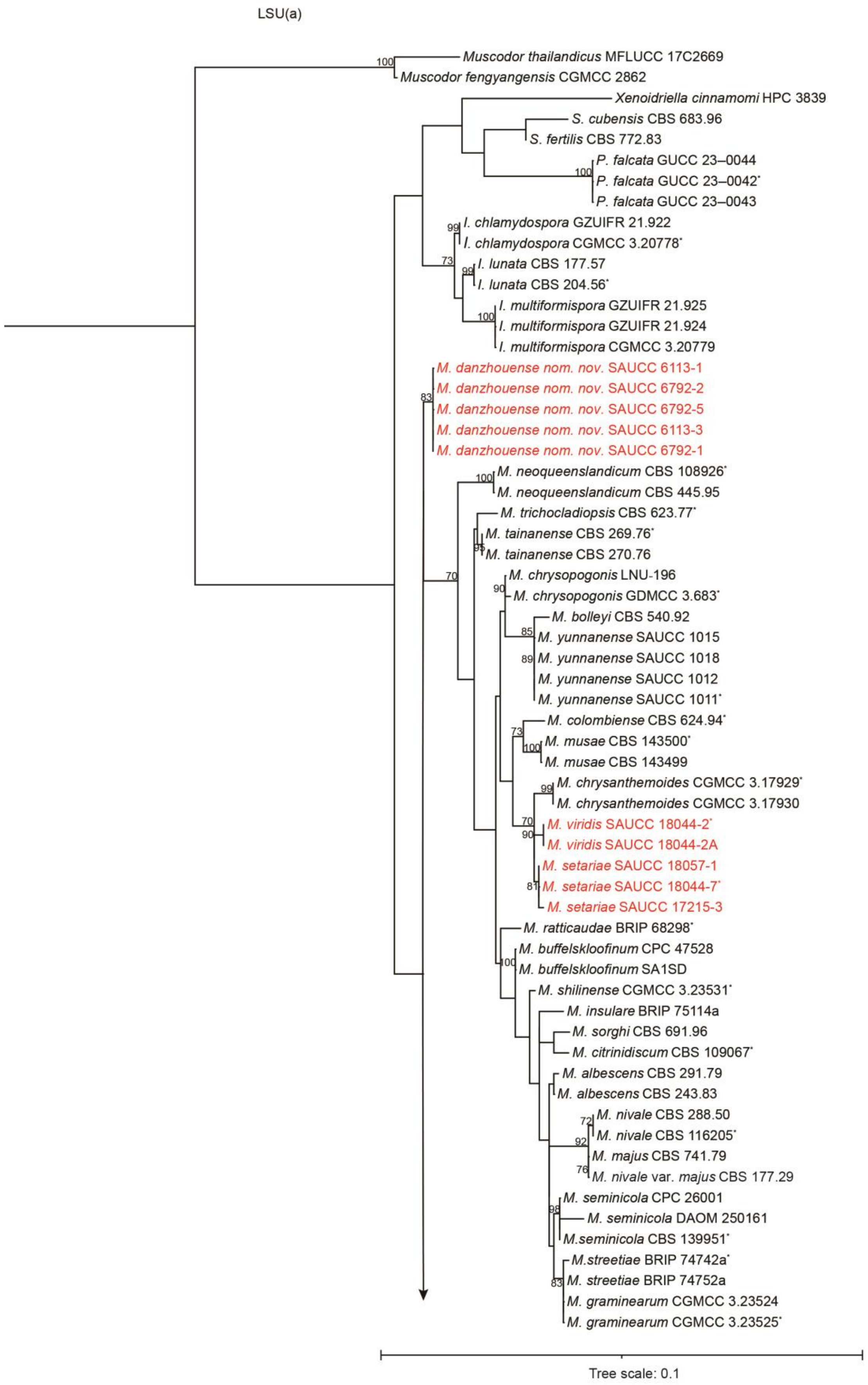
LSU(a)
Muscodor thailandicus MFLUCC 17C2669
Muscodor fengyangensis CGMCC 2862
Xenoidriella cinnamomi HPC 3839
S. cubensis CBS 683.96
S. fertilis CBS 772.83
P. falcata GUCC 23–0044
P. falcata GUCC 23–0042*
P. falcata GUCC 23–0043
I. chlamydospora GZUIFR 21.922
I. chlamydospora CGMCC 3.20778*
I. lunata CBS 177.57
I. lunata CBS 204.56*
I. multiformispora GZUIFR 21.925
I. multiformispora GZUIFR 21.924
I. multiformispora CGMCC 3.20779
M. danzhouense nom. nov. SAUCC 6113-1
M. danzhouense nom. nov. SAUCC 6792-2
M. danzhouense nom. nov. SAUCC 6792-5
M. danzhouense nom. nov. SAUCC 6113-3
M. danzhouense nom. nov. SAUCC 6792-1
M. neoqueenslandicum CBS 108926*
M. neoqueenslandicum CBS 445.95
M. trichocladiopsis CBS 623.77*
M. tainanense CBS 269.76*
M. tainanense CBS 270.76
M. chrysopogonis LNU-196
M. chrysopogonis GDMCC 3.683*
M. bolleyi CBS 540.92
M. yunnanense SAUCC 1015
M. yunnanense SAUCC 1018
M. yunnanense SAUCC 1012
M. yunnanense SAUCC 1011*
M. colombiense CBS 624.94*
M. musae CBS 143500*
M. musae CBS 143499
M. chrysanthemoides CGMCC 3.17929*
M. chrysanthemoides CGMCC 3.17930
M. viridis SAUCC 18044-2*
M. viridis SAUCC 18044-2A
M. setariae SAUCC 18057-1
M. setariae SAUCC 18044-7*
M. setariae SAUCC 17215-3
M. ratticaudae BRIP 68298*
M. buffelskloofinum CPC 47528
M. buffelskloofinum SA1SD
M. shilinense CGMCC 3.23531*
M. insulare BRIP 75114a
M. sorghi CBS 691.96
M. citrinidiscum CBS 109067*
M. albescens CBS 291.79
M. albescens CBS 243.83
M. nivale CBS 288.50
M. nivale CBS 116205*
M. majus CBS 741.79
M. nivale var. majus CBS 177.29
M. seminicola CPC 26001
M. seminicola DAOM 250161
M.seminicola CBS 139951*
M.streetiae BRIP 74742a*
M. streetiae BRIP 74752a
M. graminearum CGMCC 3.23524
M. graminearum CGMCC 3.23525*
Tree scale: 0.1

LSU(b)

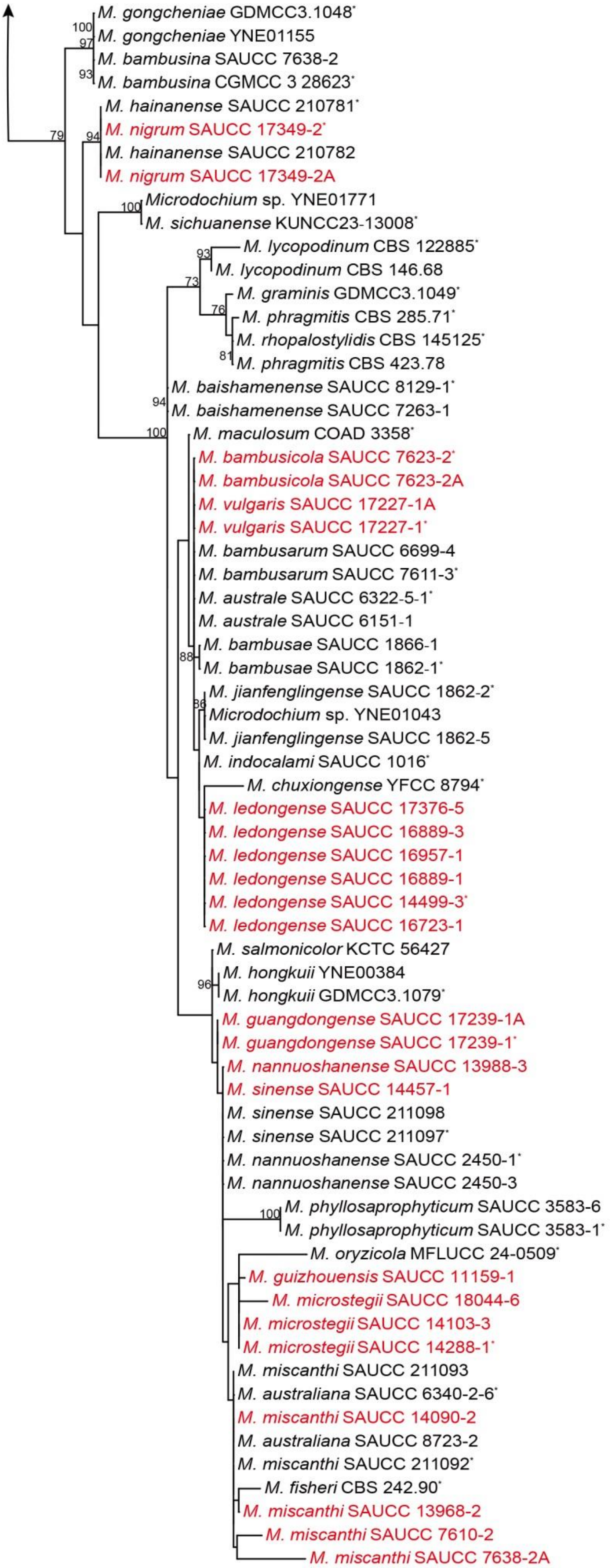
M. gongcheniae GDMCC3.1048*
M. gongcheniae YNE01155
M. bambusina SAUCC 7638-2
M. bambusina CGMCC 3 28623*
M. hainanense SAUCC 210781*
M. nigrum SAUCC 17349-2*
M. hainanense SAUCC 210782
M. nigrum SAUCC 17349-2A
Microdochium sp. YNE01771
M. sichuanense KUNCC23-13008*
M. lycopodinum CBS 122885*
M. lycopodinum CBS 146.68
M. graminis GDMCC3.1049*
M. phragmitis CBS 285.71*
M. rhopalostylidis CBS 145125*
M. phragmitis CBS 423.78
M. baishamenense SAUCC 8129-1*
M. baishamenense SAUCC 7263-1
M. maculosum COAD 3358*
M. bambusicola SAUCC 7623-2*
M. bambusicola SAUCC 7623-2A
M. vulgaris SAUCC 17227-1A
M. vulgaris SAUCC 17227-1*
M. bambusarum SAUCC 6699-4
M. bambusarum SAUCC 7611-3*
M. australe SAUCC 6322-5-1*
M. australe SAUCC 6151-1
M. bambusae SAUCC 1866-1
M. bambusae SAUCC 1862-1*
M. jianfenglingense SAUCC 1862-2*
Microdochium sp. YNE01043
M. jianfenglingense SAUCC 1862-5
M. indocalami SAUCC 1016*
M. chuxiongense YFCC 8794*
M. ledongense SAUCC 17376-5
M. ledongense SAUCC 16889-3
M. ledongense SAUCC 16957-1
M. ledongense SAUCC 16889-1
M. ledongense SAUCC 14499-3*
M. ledongense SAUCC 16723-1
M. salmonicolor KCTC 56427
M. hongkuii YNE00384
M. hongkuii GDMCC3.1079*
M. guangdongense SAUCC 17239-1A
M. guangdongense SAUCC 17239-1*
M. nannuoshanense SAUCC 13988-3
M. sinense SAUCC 14457-1
M. sinense SAUCC 211098
M. sinense SAUCC 211097*
M. nannuoshanense SAUCC 2450-1*
M. nannuoshanense SAUCC 2450-3
M. phyllosaprophyticum SAUCC 3583-6
M. phyllosaprophyticum SAUCC 3583-1*
M. oryzicola MFLUCC 24-0509*
M. guizhouensis SAUCC 11159-1
M. microstegii SAUCC 18044-6
M. microstegii SAUCC 14103-3
M. microstegii SAUCC 14288-1*
M. miscanthi SAUCC 211093
M. australiana SAUCC 6340-2-6*
M. miscanthi SAUCC 14090-2
M. australiana SAUCC 8723-2
M. miscanthi SAUCC 211092*
M. fisheri CBS 242.90*
M. miscanthi SAUCC 13968-2
M. miscanthi SAUCC 7610-2
M. miscanthi SAUCC 7638-2A

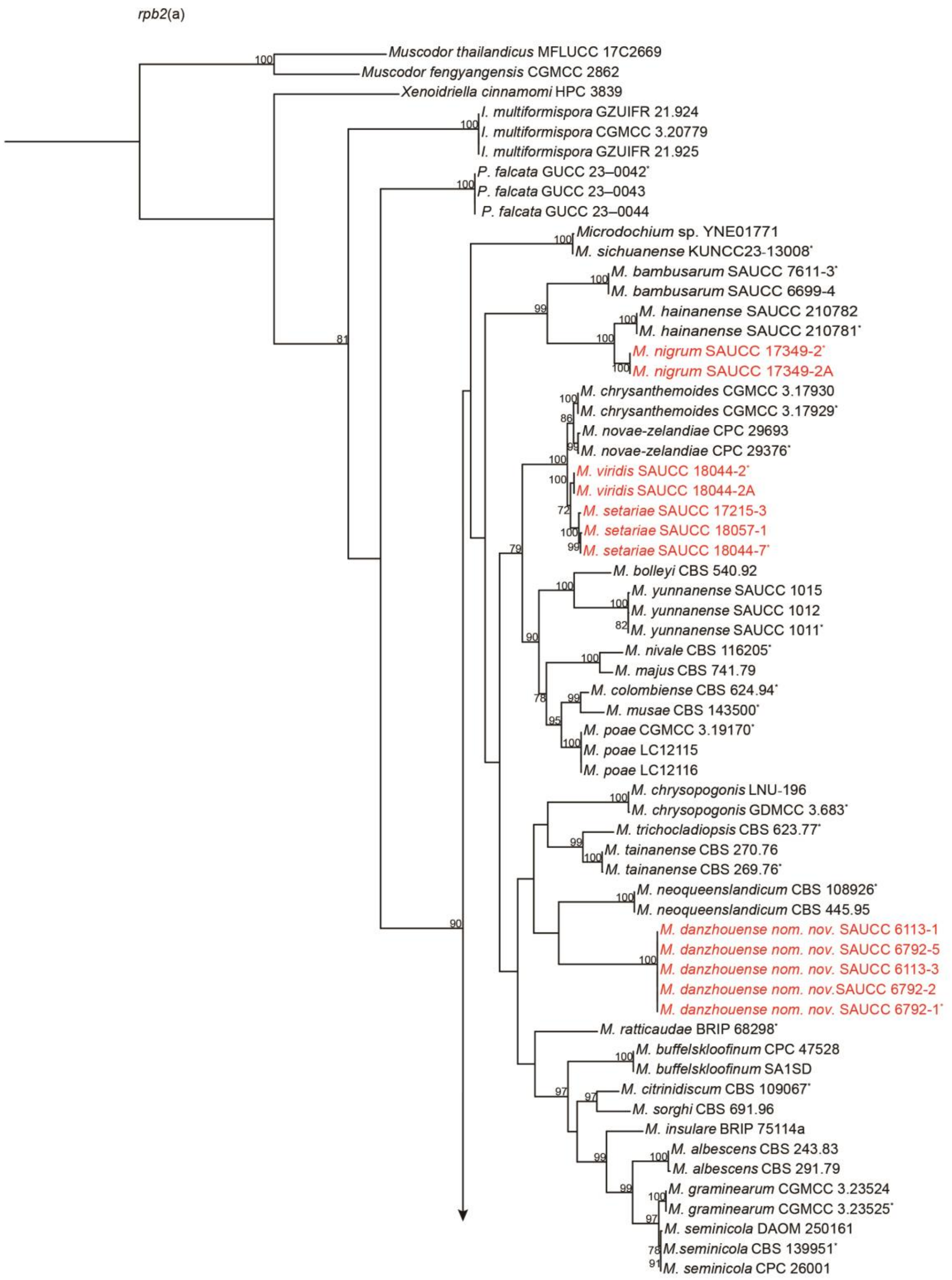
rpb2(a)
Muscodor thailandicus MFLUCC 17C2669
Muscodor fengyangensis CGMCC 2862
Xenoidriella cinnamomi HPC 3839
I. multiformispora GZUIFR 21.924
I. multiformispora CGMCC 3.20779
I. multiformispora GZUIFR 21.925
P. falcata GUCC 23–0042*
P. falcata GUCC 23–0043
P. falcata GUCC 23–0044
Microdochium sp. YNE01771
M. sichuanense KUNCC23-13008*
M. bambusarum SAUCC 7611-3*
M. bambusarum SAUCC 6699-4
M. hainanense SAUCC 210782
M. hainanense SAUCC 210781*
M. nigrum SAUCC 17349-2*
M. nigrum SAUCC 17349-2A
M. chrysanthemoides CGMCC 3.17930
M. chrysanthemoides CGMCC 3.17929*
M. novae-zelandiae CPC 29693
M. novae-zelandiae CPC 29376*
M. viridis SAUCC 18044-2*
M. viridis SAUCC 18044-2A
M. setariae SAUCC 17215-3
M. setariae SAUCC 18057-1
M. setariae SAUCC 18044-7*
M. bolleyi CBS 540.92
M. yunnanense SAUCC 1015
M. yunnanense SAUCC 1012
M. yunnanense SAUCC 1011*
M. nivale CBS 116205*
M. majus CBS 741.79
M. colombiense CBS 624.94*
M. musae CBS 143500*
M. poae CGMCC 3.19170*
M. poae LC12115
M. poae LC12116
M. chrysopogonis LNU-196
M. chrysopogonis GDMCC 3.683*
M. trichocladiopsis CBS 623.77*
M. tainanense CBS 270.76
M. tainanense CBS 269.76*
M. neoqueenslandicum CBS 108926*
M. neoqueenslandicum CBS 445.95
M. danzhouense nom. nov. SAUCC 6113-1
M. danzhouense nom. nov. SAUCC 6792-5
M. danzhouense nom. nov. SAUCC 6113-3
M. danzhouense nom. nov.SAUCC 6792-2
M. danzhouense nom. nov. SAUCC 6792-1*
M. ratticaudae BRIP 68298*
M. buffelskloofinum CPC 47528
M. buffelskloofinum SA1SD
M. citrinidiscum CBS 109067*
M. sorghi CBS 691.96
M. insulare BRIP 75114a
M. albescens CBS 243.83
M. albescens CBS 291.79
M. graminearum CGMCC 3.23524
M. graminearum CGMCC 3.23525*
M. seminicola DAOM 250161
M.seminicola CBS 139951*
M. seminicola CPC 26001

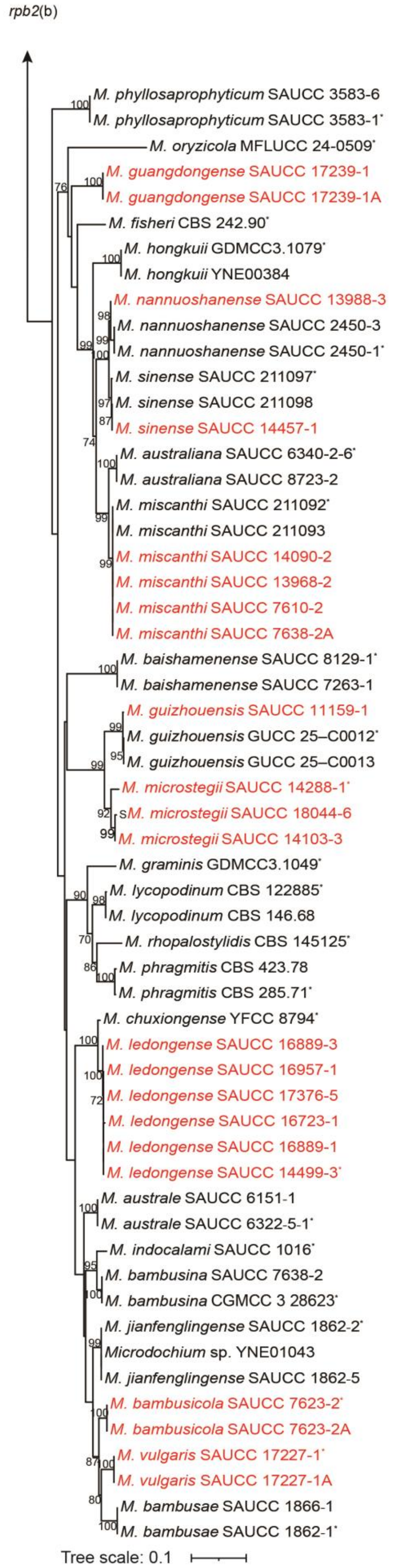

rpb2(b)
M. phyllosaprophyticum SAUCC 3583-6
M. phyllosaprophyticum SAUCC 3583-1*
M. oryzicola MFLUCC 24-0509*
M. guangdongense SAUCC 17239-1
M. guangdongense SAUCC 17239-1A
M. fisheri CBS 242.90*
M. hongkuii GDMCC3.1079*
M. hongkuii YNE00384
M. nannuoshanense SAUCC 13988-3
M. nannuoshanense SAUCC 2450-3
M. nannuoshanense SAUCC 2450-1*
M. sinense SAUCC 211097*
M. sinense SAUCC 211098
M. sinense SAUCC 14457-1
M. australiana SAUCC 6340-2-6*
M. australiana SAUCC 8723-2
M. miscanthi SAUCC 211092*
M. miscanthi SAUCC 211093
M. miscanthi SAUCC 14090-2
M. miscanthi SAUCC 13968-2
M. miscanthi SAUCC 7610-2
M. miscanthi SAUCC 7638-2A
M. baishamenense SAUCC 8129-1*
M. baishamenense SAUCC 7263-1
M. guizhouensis SAUCC 11159-1
M. guizhouensis GUCC 25–C0012*
M. guizhouensis GUCC 25–C0013
M. microstegii SAUCC 14288-1*
M. microstegii SAUCC 18044-6
M. microstegii SAUCC 14103-3
M. graminis GDMCC3.1049*
M. lycopodinum CBS 122885*
M. lycopodinum CBS 146.68
M. rhopalostylidis CBS 145125*
M. phragmitis CBS 423.78
M. phragmitis CBS 285.71*
M. chuxiongense YFCC 8794*
M. ledongense SAUCC 16889-3
M. ledongense SAUCC 16957-1
M. ledongense SAUCC 17376-5
M. ledongense SAUCC 16723-1
M. ledongense SAUCC 16889-1
M. ledongense SAUCC 14499-3*
M. australe SAUCC 6151-1
M. australe SAUCC 6322-5-1*
M. indocalami SAUCC 1016*
M. bambusina SAUCC 7638-2
M. bambusina CGMCC 3 28623*
M. jianfenglingense SAUCC 1862-2*
Microdochium sp. YNE01043
M. jianfenglingense SAUCC 1862-5
M. bambusicola SAUCC 7623-2*
M. bambusicola SAUCC 7623-2A
M. vulgaris SAUCC 17227-1*
M. vulgaris SAUCC 17227-1A
M. bambusae SAUCC 1866-1
M. bambusae SAUCC 1862-1*
Tree scale: 0.1

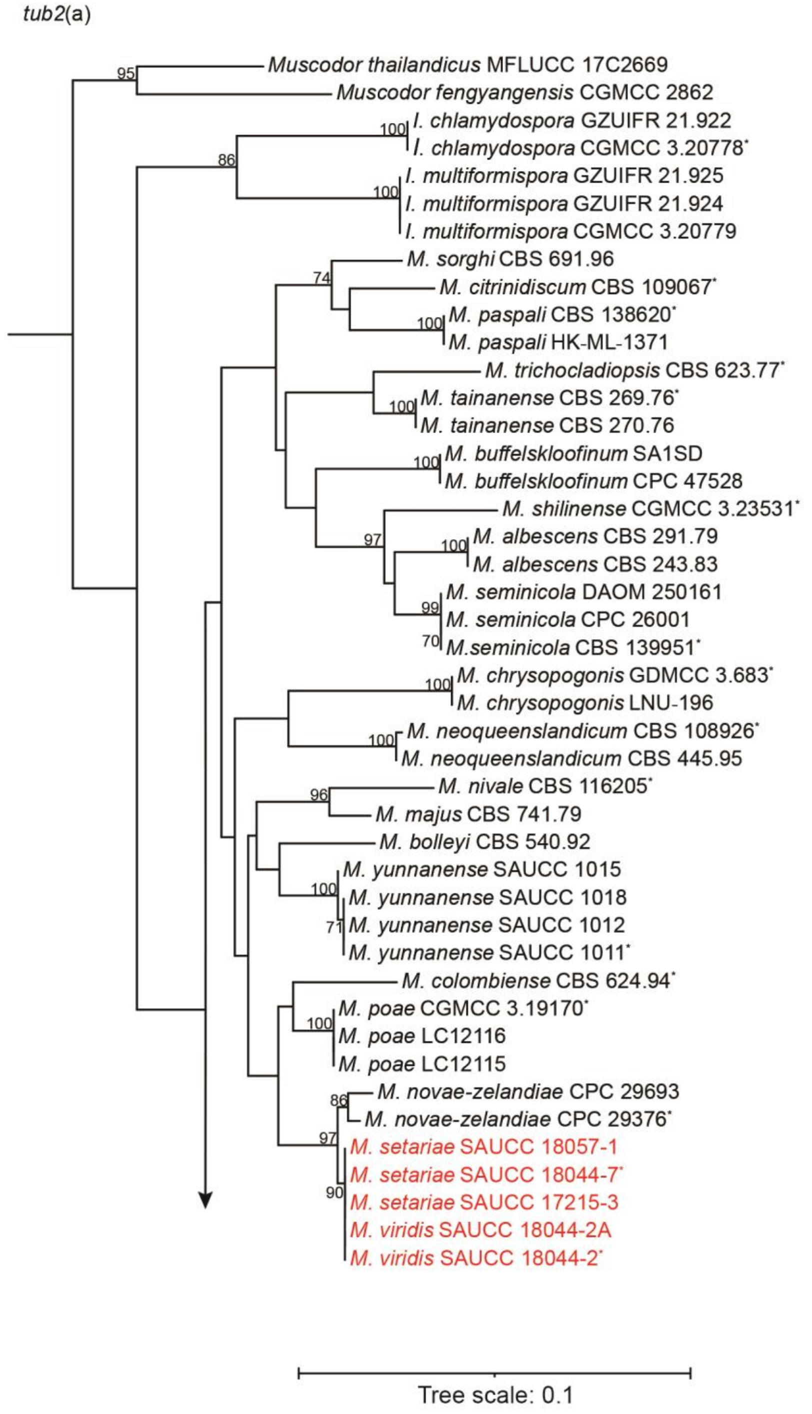
tub2(a)
Muscodor thailandicus MFLUCC 17C2669
Muscodor fengyangensis CGMCC 2862
I. chlamydospora GZUIFR 21.922
I. chlamydospora CGMCC 3.20778*
I. multiformispora GZUIFR 21.925
I. multiformispora GZUIFR 21.924
I. multiformispora CGMCC 3.20779
M. sorghi CBS 691.96
M. citrinidiscum CBS 109067*
M. paspali CBS 138620*
M. paspali HK-ML-1371
M. trichocladiopsis CBS 623.77*
M. tainanense CBS 269.76*
M. tainanense CBS 270.76
M. buffelskloofinum SA1SD
M. buffelskloofinum CPC 47528
M. shilinense CGMCC 3.23531*
M. albescens CBS 291.79
M. albescens CBS 243.83
M. seminicola DAOM 250161
M. seminicola CPC 26001
M.seminicola CBS 139951*
M. chrysopogonis GDMCC 3.683*
M. chrysopogonis LNU-196
M. neoqueenslandicum CBS 108926*
M. neoqueenslandicum CBS 445.95
M. nivale CBS 116205*
M. majus CBS 741.79
M. bolleyi CBS 540.92
M. yunnanense SAUCC 1015
M. yunnanense SAUCC 1018
M. yunnanense SAUCC 1012
M. yunnanense SAUCC 1011*
M. colombiense CBS 624.94*
M. poae CGMCC 3.19170*
M. poae LC12116
M. poae LC12115
M. novae-zelandiae CPC 29693
M. novae-zelandiae CPC 29376*
M. setariae SAUCC 18057-1
M. setariae SAUCC 18044-7*
M. setariae SAUCC 17215-3
M. viridis SAUCC 18044-2A
M. viridis SAUCC 18044-2*
Tree scale: 0.1

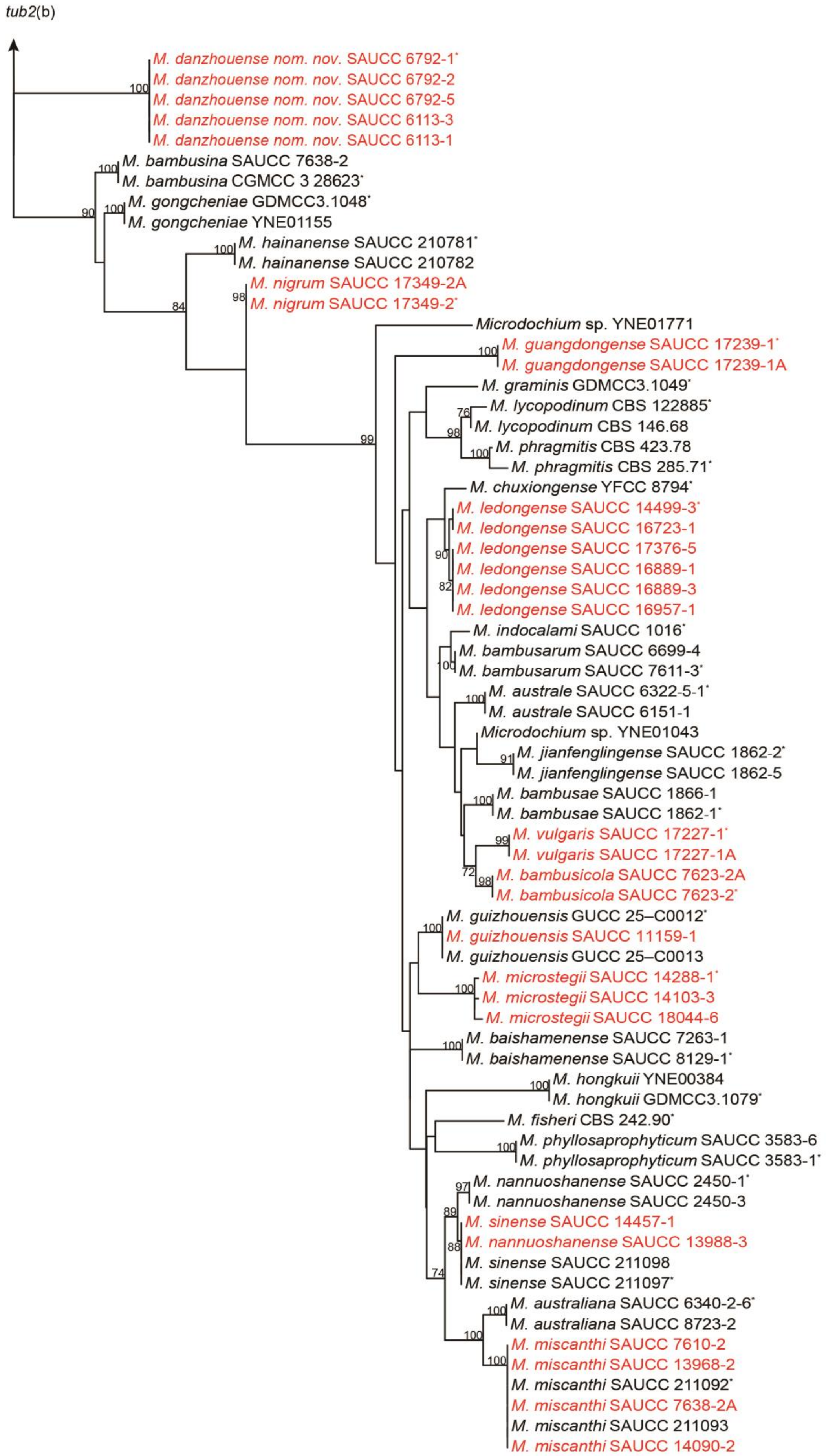
tub2(b)
M. danzhouense nom. nov. SAUCC 6792-1*
M. danzhouense nom. nov. SAUCC 6792-2
M. danzhouense nom. nov. SAUCC 6792-5
M. danzhouense nom. nov. SAUCC 6113-3
M. danzhouense nom. nov. SAUCC 6113-1
M. bambusina SAUCC 7638-2
M. bambusina CGMCC 3 28623*
M. gongcheniae GDMCC3.1048*
M. gongcheniae YNE01155
M. hainanense SAUCC 210781*
M. hainanense SAUCC 210782
M. nigrum SAUCC 17349-2A
M. nigrum SAUCC 17349-2*
Microdochium sp. YNE01771
M. guangdongense SAUCC 17239-1*
M. guangdongense SAUCC 17239-1A
M. graminis GDMCC3.1049*
M. lycopodinum CBS 122885*
M. lycopodinum CBS 146.68
M. phragmitis CBS 423.78
M. phragmitis CBS 285.71*
M. chuxiongense YFCC 8794*
M. ledongense SAUCC 14499-3*
M. ledongense SAUCC 16723-1
M. ledongense SAUCC 17376-5
M. ledongense SAUCC 16889-1
M. ledongense SAUCC 16889-3
M. ledongense SAUCC 16957-1
M. indocalami SAUCC 1016*
M. bambusarum SAUCC 6699-4
M. bambusarum SAUCC 7611-3*
M. australe SAUCC 6322-5-1*
M. australe SAUCC 6151-1
Microdochium sp. YNE01043
M. jianfenglingense SAUCC 1862-2*
M. jianfenglingense SAUCC 1862-5
M. bambusae SAUCC 1866-1
M. bambusae SAUCC 1862-1*
M. vulgaris SAUCC 17227-1*
M. vulgaris SAUCC 17227-1A
M. bambusicola SAUCC 7623-2A
M. bambusicola SAUCC 7623-2*
M. guizhouensis GUCC 25–C0012*
M. guizhouensis SAUCC 11159-1
M. guizhouensis GUCC 25–C0013
M. microstegii SAUCC 14288-1*
M. microstegii SAUCC 14103-3
M. microstegii SAUCC 18044-6
M. baishamenense SAUCC 7263-1
M. baishamenense SAUCC 8129-1*
M. hongkuii YNE00384
M. hongkuii GDMCC3.1079*
M. fisheri CBS 242.90*
M. phyllosaprophyticum SAUCC 3583-6
M. phyllosaprophyticum SAUCC 3583-1*
M. nannuoshanense SAUCC 2450-1*
M. nannuoshanense SAUCC 2450-3
M. sinense SAUCC 14457-1
M. nannuoshanense SAUCC 13988-3
M. sinense SAUCC 211098
M. sinense SAUCC 211097*
M. australiana SAUCC 6340-2-6*
M. australiana SAUCC 8723-2
M. miscanthi SAUCC 7610-2
M. miscanthi SAUCC 13968-2
M. miscanthi SAUCC 211092*
M. miscanthi SAUCC 7638-2A
M. miscanthi SAUCC 211093
M. miscanthi SAUCC 14090-2
100
100
90
100
100
84
98
99
100
76
98
100
90
82
100
100
91
100
99
72
98
100
100
100
100
100
97
89
88
74
100
100
100

Supplementary material 2: Single-locus phylograms of the family *Microdochiaceae* based on alignments of the ITS, LSU, *rpb2*, and *tub2* regions, respectively. Each tree is rooted with *Muscodor fengyangensis* (CGMCC 2862) and *Muscodor thailandicus* (MFLUCC 17–2669) as the outgroups. The Maximum Likelihood Bootstrap Value (MLBV ≥ 70%) are shown above the nodes. Strains isolated in this study were indicated in red.
